# Joint representation of spliced and unspliced RNA expands information content in single-cell expression data

**DOI:** 10.1093/bioadv/vbag263

**Published:** 2026-10-22

**Authors:** Arya Jadhav, Zachary J DeBruine

**Affiliations:** Department of Information Sciences and Technology, Grand Valley State University, Grand Rapids, MI 49504, United States; Department of Information Sciences and Technology, Grand Valley State University, Grand Rapids, MI 49504, United States

## Abstract

**Motivation:**

Single-cell RNA-seq captures both mature (spliced) and nascent (unspliced) transcripts, yet standard preprocessing collapses these signals into a single expression matrix, obscuring recoverable structure and introducing splicing-dependent artifacts in downstream analysis. We asked whether treating spliced and unspliced counts as distinct feature sets increases recoverable latent structure in unsupervised dimension reduction.

**Results:**

Across four datasets, we applied non-negative matrix factorization to spliced-only, unspliced-only, added, and concatenated representations, estimating the revealable rank, the largest stable rank achieving near-optimal held-out error, by cross-validation. Concatenated representations exceeded the added representation in all four datasets and surpassed both single-modality inputs in three of four, indicating that preserving splicing status exposes additional stable latent programs beyond either modality alone or their sum. Minimal overlap between spliced- and unspliced-derived factors corresponded to modality-specific gene set enrichment consistent with complementary steady-state and nascent regulatory activity. These results establish splicing status as an informative axis of variation and motivate preserving spliced and unspliced layers as first-class features to maximize information recovery.

**Availability and Implementation:**

The singlet R package is available at github.com/zdebruine/singlet. Code to reproduce all figures is available at github.com/Arya-86/nmf-spliced-unspliced.

## 1 Introduction

Single-cell RNA sequencing (scRNA-seq) has become a cornerstone technology for exploring cellular diversity and gene regulation at single-cell resolution. Its adoption has accelerated through large-scale initiatives such as the Human Cell Atlas (Regev *et al*. 2017) and the BRAIN Initiative Cell Census Consortium (Ecker *et al*. 2017), which are generating reference atlases that will shape the next decade of transcriptomic discovery. As these datasets expand in scale and resolution, analytical conventions governing how gene expression is quantified and reduced have a profound influence on what biological structure can be revealed (Hemberg *et al*. 2025).

In most workflows, sequencing reads are aligned to a reference transcriptome to quantify spliced, mature mRNA molecules. The resulting gene expression matrix therefore reflects the abundance of processed transcripts that have completed splicing and export. However, single-cell sequencing protocols also capture a substantial proportion of unspliced, intron-containing pre-mRNAs (La Manno *et al*. 2018). These reads originate from nascent transcripts within the nucleus and carry information about genes currently undergoing transcriptional activation. Despite their ubiquity, unspliced reads are often discarded or summed with spliced reads, erasing distinctions between transcriptional activity and steady-state expression. This convention emerged largely from computational convenience and compatibility with downstream tools rather than empirical evidence of redundancy between the two signals (Luecken and Theis 2019).

The treatment of spliced and unspliced reads in scRNA-seq quantification has evolved markedly over the past decade. Early single-cell pipelines restricted quantification to reads mapping to exons, assuming that exon-aligned reads best represent functional mRNA abundance. With the advent of single-nucleus RNA-seq (snRNA-seq) and dynamic modeling frameworks, inclusion of intronic reads has become increasingly common. In such datasets, intronic reads can account for 20%–50% of total unique molecular identifiers (UMIs) (Bakken *et al*. 2018). Modern quantification pipelines such as 10× Genomics Cell Ranger (v7.0 and later) and STARsolo now default to whole-transcriptome quantification, aligning reads to both exons and introns to maximize sensitivity and preserve information from both transcriptional compartments (Kaminow *et al*. 2021, 10x Genomics 2022). This shift effectively redefines the standard measure of “gene expression” in scRNA-seq from cytoplasmic mRNA abundance to a composite signal reflecting total transcriptional output, even though intronic and exonic reads are typically aggregated.

Recent long-read single-cell studies confirm that gene-level aggregation can mask biologically meaningful transcript-level structure: Chen *et al*. (2026) showed that isoform-level analysis with IsoDiffR reveals cell-type-specific expression patterns invisible to gene-level approaches, reinforcing the cost of collapsing molecularly distinct transcript classes.

The most influential framework exploiting splicing information is RNA velocity (La Manno *et al*. 2018, Bergen *et al*. 2020). RNA velocity estimates the future transcriptional state of a cell by comparing spliced and unspliced transcript abundances, modeling the kinetics of transcription, splicing, and degradation. Velocity analysis depends on constructing separate spliced and unspliced count matrices using specialized quantification tools such as velocyto (La Manno *et al*. 2018), alevin-fry (He *et al*. 2022), or the velocity feature of STARsolo (Kaminow *et al*. 2021), which are now distributed as part of standard pipelines. While RNA velocity has produced compelling perspectives on cellular differentiation and lineage trajectories, it relies on simplifying assumptions regarding kinetic steady state, uniform splicing rates, and accurate classification of transcript status, which may introduce variability across datasets (Bergen *et al*. 2020, Gorin *et al*. 2022). Benchmark studies further demonstrate that velocity estimates can differ substantially depending on quantification software and parameterization (He *et al*. 2022, Schleicher *et al*. 2026).

Nevertheless, the widespread adoption of RNA velocity has established a robust infrastructure for generating and storing spliced and unspliced matrices as standard outputs of single-cell pipelines. This availability enables direct multivariate analysis of these modalities as complementary inputs for exploratory analysis. Treating spliced and unspliced counts as distinct yet interrelated features may reveal additional structure in the data without invoking kinetic modeling.

This framing is consistent with broader evidence that single-cell representations benefit from modeling complementary structure rather than a single collapsed view (Li *et al*. 2025), supporting the principle that preserving biologically distinct information sources enhances downstream structure recovery.

The present study is motivated by a foundational question that remains unexplored despite the ubiquity of spliced and unspliced data: does treating spliced and unspliced transcripts as independent, first-class features enhance the latent structure recoverable from single-cell expression matrices? If these transcript classes encode complementary facets of gene regulation, then modeling both explicitly should increase the effective dimensionality and explainability of low-rank representations.

To investigate this question, we employ a cross-validated, unsupervised factorization framework based on Non-negative Matrix Factorization (NMF) (Lee and Seung 1999, Guo *et al*. 2024). NMF provides an interpretable, additive decomposition well-suited to non-negative count-based biological data and allows direct evaluation of reconstruction fidelity and rank stability. By applying this framework to public scRNA-seq datasets quantified for both spliced and unspliced reads, we assess how these transcript classes jointly define the expressible subspace of transcriptional variation, extending splicing-aware analysis beyond trajectory inference toward a more general characterization of transcriptional structure.

## 2 Results

### 2.1 Revealable rank provides an interpretable measure of information content across feature representations

Single-cell RNA sequencing captures both mature (spliced) and nascent (unspliced) RNA molecules (La Manno *et al*. 2018, Bergen *et al*. 2020). Conventional workflows often collapse these signals into a single expression matrix, implicitly treating them as redundant. However, spliced and unspliced counts reflect different transcriptional processes and may therefore encode complementary biological information (Gorin *et al*. 2022). To test this hypothesis, we analysed paired spliced and unspliced count layers across multiple datasets under four feature representations: spliced-only, unspliced-only, added (spliced + unspliced), and concatenated (spliced ⊕ unspliced) features (Fig. 1).

**Figure 1:**
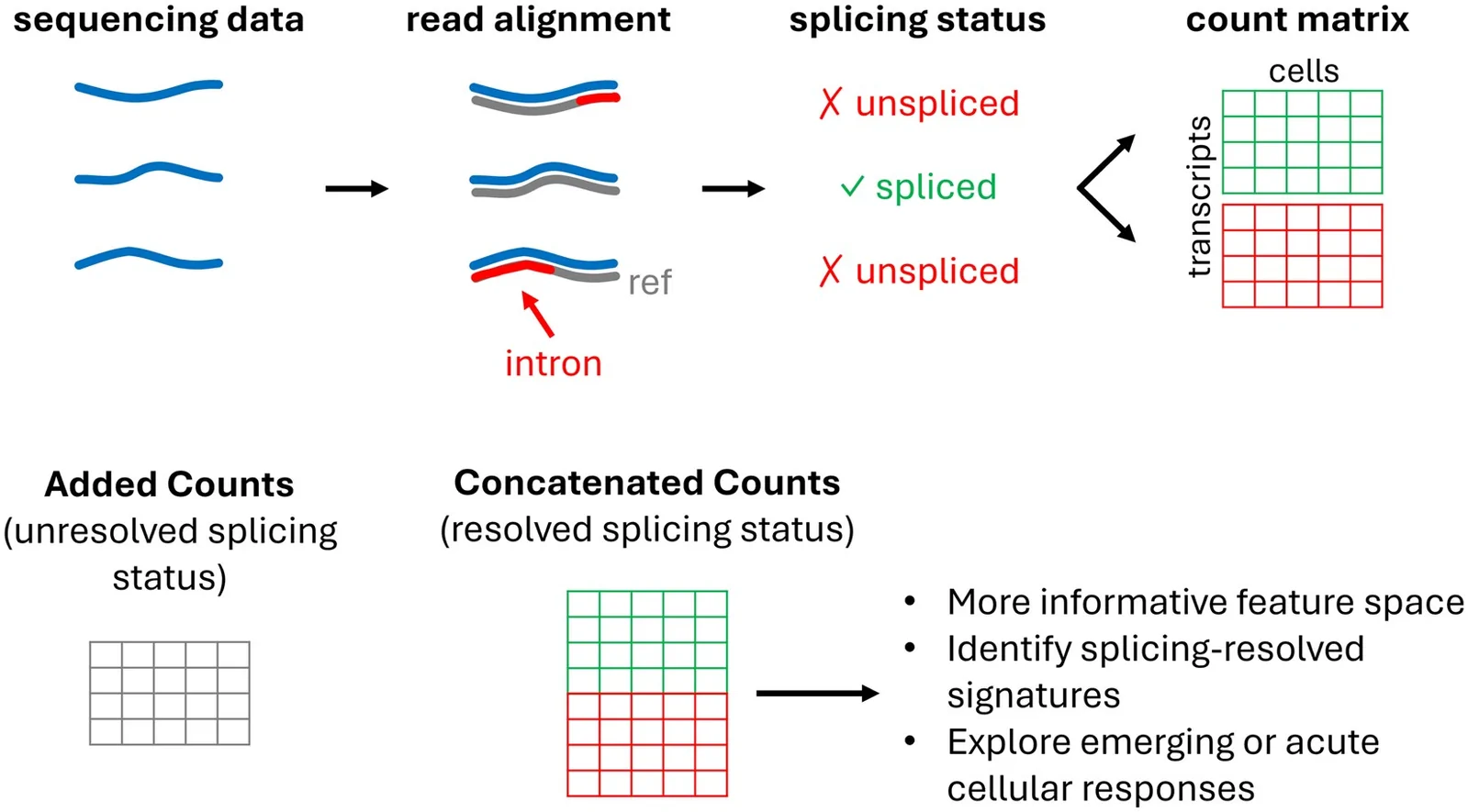
Conceptual overview of spliced and unspliced quantification in single-cell RNA-seq. Single-cell workflows typically align reads to transcriptomic references that do not include intronic regions, producing a count matrix of transcripts × cells that conflates spliced and unspliced molecules. When alignment includes intronic annotation, the splicing state of each read can be resolved, yielding two matrices—spliced and unspliced—that can be concatenated into a joint representation. This joint feature space provides a richer foundation for dimensionality reduction and integrative modeling of cellular state than standard pre-processing that adds spliced and unspliced reads together.

To quantify how feature representation affects the amount of structure recoverable from the data, we determine the revealable rank $k^{*}$: the largest factorization rank that can be learned stably without overfitting, as assessed by cross-validation (Wold 1978, Owen and Perry 2009). Intuitively, $k^{*}$ approximates the effective dimensionality of the transcriptional signal supported by a given representation, or in other words, the number of distinct latent programs that can be reconstructed in a way that generalizes beyond the observed entries. We treat $k^{*}$ as an operational proxy for recoverable information content rather than a direct information-theoretic measure; it quantifies how many stable, generalizable factors a representation supports under cross-validation (Tunnell *et al*. 2025).

We estimate $k^{*}$ by fitting NMF across a span of ranks and tracking held-out reconstruction error as model capacity increases. As illustrated in Fig. 2B, low ranks underfit, intermediate ranks achieve stable minima, and sufficiently large ranks overfit. We take $k^{*}$ as the largest rank within the near-minimum plateau of the cross-validation curve, immediately preceding sustained overfitting, as described in Methods. In the following section, we apply this framework uniformly to the four feature representations.

**Figure 2:**
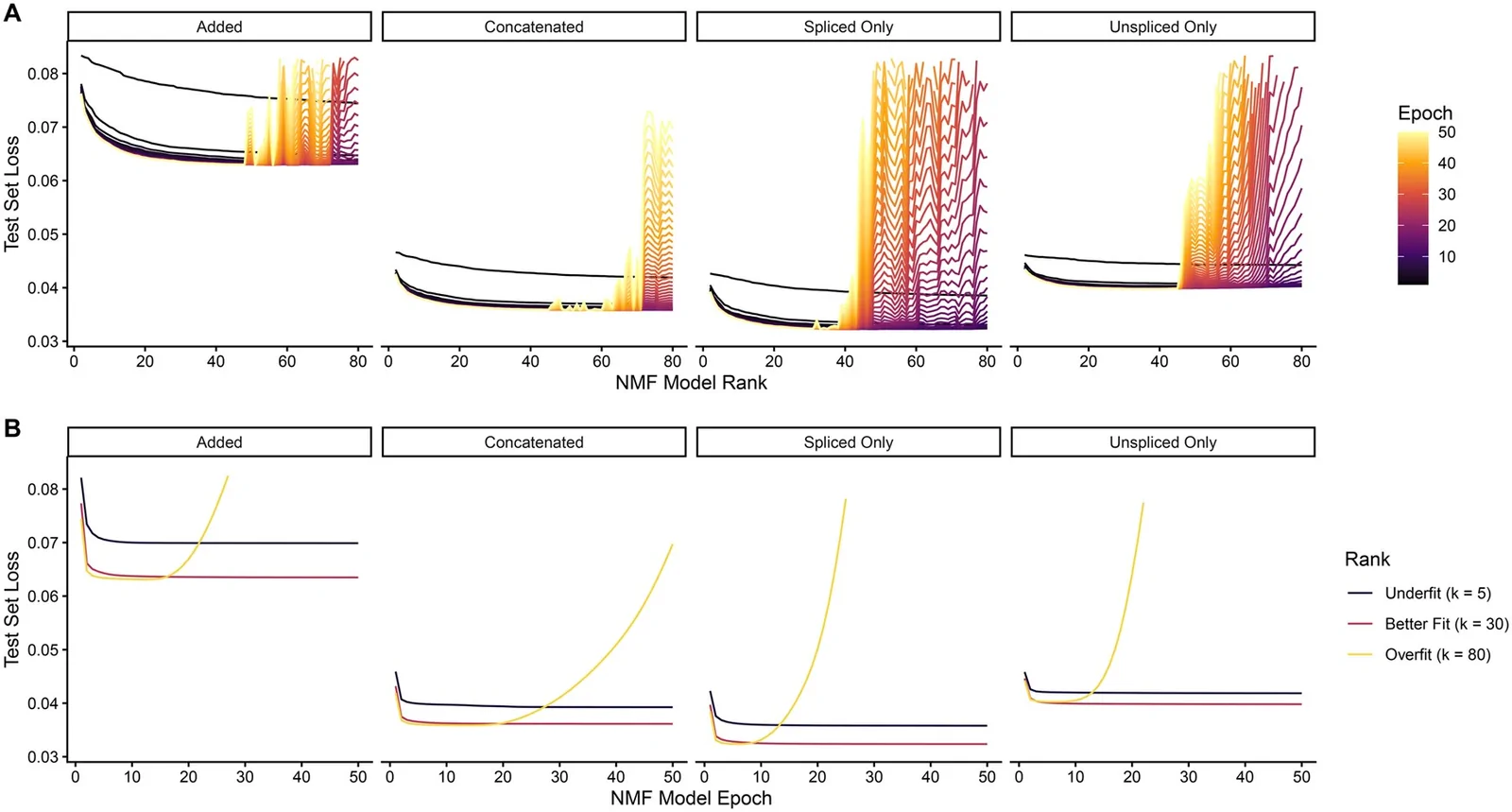
Cross-validation of NMF on the Heart dataset defines the revealable rank for each modality. (A) Mean test-set reconstruction error across NMF ranks (k=2,…,80) for spliced-only, unspliced-only, added, and concatenated inputs. Low ranks underfit, showing large unexplained variance, whereas high ranks overfit, reflected by rising test error despite improved training loss. (B) Representative trajectories illustrate underfit (k=5), approximate representative optimal (k=30), and representative overfit (k=80) behavior, demonstrating how overfitting is recognized as an instability of test loss as the model fits.

### 2.2 Concatenating spliced and unspliced features increases revealable rank

We analysed four public 10× Genomics datasets commonly used in RNA-velocity studies (Gorin *et al*. 2022) (Heart, Brain, Neuron, and PBMC) to assess how spliced/unspliced feature representation affects the recoverable latent structure. Across all four datasets and three replicates, concatenating spliced and unspliced features generally increased the revealable rank relative to models fit to spliced-only or unspliced-only inputs (Fig. 2A; Table 1; Fig. S2; Tables S1–4). The relative information content of the two modalities was dataset-dependent: in some datasets unspliced supported higher rank than spliced, whereas in others the reverse held, indicating that neither modality is uniformly dominant and both contain substantial resolvable information structure. Combining modalities increased revealable rank beyond either single layer in three of four datasets but by less than the sum of both. Concatenation yielded higher revealable rank than the added representation across all four datasets (Brain: 46.3 versus 42.7; Heart: 55.0 versus 47.7; Neuron: 75.7 versus 70.7; PBMC: 44.7 versus 42.0), indicating that preserving spliced and unspliced counts as distinct features enables NMF to resolve additional latent programs that are partially obscured when the modalities are summed. The magnitude of this advantage varied by dataset, with Heart showing the clearest separation and PBMC the most modest difference, consistent with dataset-specific variation in the relative information content of the two modalities.

**Table 1:** Optimal NMF rank based on cross-validation results (mean ± SD across three replicates).

Dataset	Spliced	Unspliced	Added	Concat
**Brain**	33.0 ± 1.0	39.7 ± 2.5	42.7 ± 2.5	46.3 ± 5.0
**Heart**	32.3 ± 1.2	48.3 ± 3.1	47.7 ± 2.5	55.0 ± 1.7
**Neuron**	63.0 ± 6.6	42.3 ± 3.1	70.7 ± 4.0	75.7 ± 3.2
**PBMC**	46.3 ± 12.9	30.3 ± 4.6	42.0 ± 1.7	44.7 ± 4.0

Concatenation yielded higher revealable rank than the added representation across all four datasets, with the advantage most pronounced in Heart (55.0 versus 47.7) and most modest in PBMC (44.7 versus 42.0). Joint representation of spliced and unspliced information consistently recovers more latent structure than either modality alone or their sum.

Principal Component Analysis (PCA) did not show a corresponding separation between spliced, unspliced, added, and concatenated representations when assessed using scree plots of marginal explained variance (Fig. S1). Unlike the cross-validated framework used to estimate revealable rank for NMF, PCA scree plots provide a descriptive heuristic and do not directly assess generalization or overfitting. A fully comparable evaluation would require a cross-validated or bi-cross-validated PCA procedure (e.g. JackStraw-style resampling) that, like our NMF analysis, assesses generalization rather than in-sample variance. We note that the absence of separation in the descriptive scree plots is expected: marginal explained variance does not penalize overfitting and therefore cannot reveal the held-out rank differences that our cross-validated criterion is designed to detect. The contrast between the two analyses is thus consistent rather than contradictory, and a cross-validated PCA comparison is a natural confirmatory analysis for future work. Accordingly, we restrict PCA comparisons to the scree plot and emphasize that the rank expansion observed for concatenated representations arises under an explicitly cross-validated factorization framework.

We further note that the rank increase under concatenation cannot be attributed to feature-count inflation alone. Although the concatenated matrix contains twice as many rows as the added matrix, two lines of evidence argue against a purely dimensional explanation. First, cross-validation penalizes uninformative capacity directly: adding rows that merely duplicate or dilute existing signal raises held-out error at a given rank rather than extending the stable region, so additional features cannot increase $k^{*}$ unless they carry generalizable structure. Second, the factor-level analyses that follow show that the additional concatenated factors are populated by genes with strongly modality-skewed loadings and distinct functional enrichment (Figs. 4 and 5), rather than redundant or noise-like dimensions—consistent with genuine biological signal rather than surplus parameters driving the rank expansion. Together these observations indicate that concatenation increases revealable rank by exposing complementary biological structure rather than by supplying additional model capacity alone.

### 2.3 Increased revealable rank reflects complementary latent structure between spliced and unspliced features

To determine whether the increase in revealable rank arises from genuinely complementary structure rather than redundant signal, we quantified the similarity between latent factors learned under each feature representation. We computed pairwise cosine similarity between NMF factors derived from spliced-only, unspliced-only, added, and concatenated inputs. Factors learned from spliced and unspliced data showed minimal overlap (Figs. 3A and S3A), indicating that the two modalities capture largely distinct transcriptional programs.

**Figure 3:**
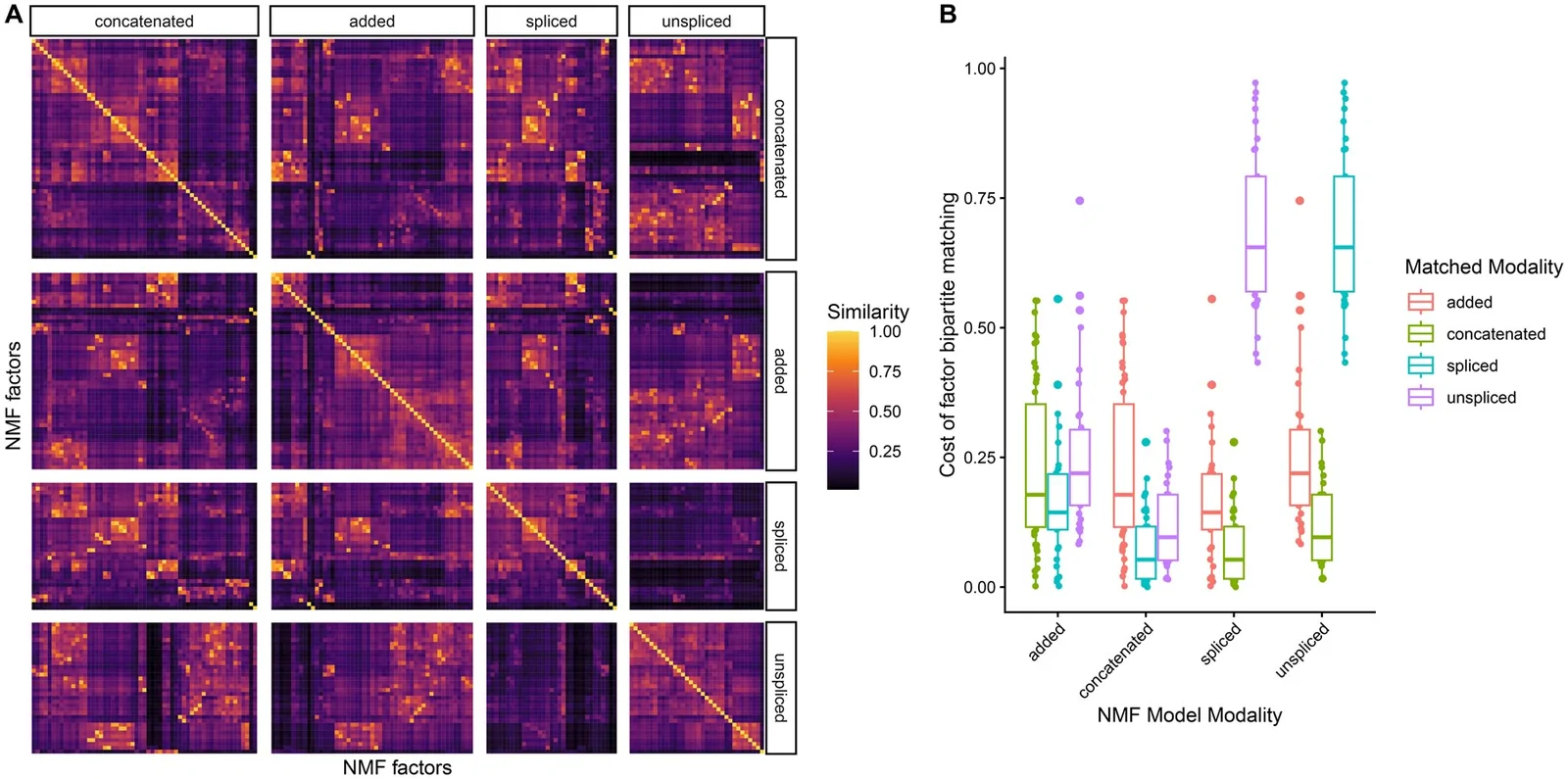
Spliced and unspliced modalities encode complementary latent structure. (A) Pairwise cosine similarity between NMF factors learned from spliced-only, unspliced-only, added, and concatenated inputs. Factors derived from spliced and unspliced data from the heart dataset exhibit minimal overlap, indicating largely non-redundant structure. (B) Optimal bipartite matching of factors across models from the heart dataset based on the similarity matrix in (A). Factors from the concatenated model show stronger correspondence to both spliced- and unspliced-derived factors than those from the added model, demonstrating that concatenation preserves modality-specific latent programs that are partially obscured by summation.

Both added and concatenated models exhibited partial correspondence to factors learned from the individual modalities. However, optimal bipartite matching revealed a clear distinction between these representations: factors from the concatenated model matched more closely to both spliced- and unspliced-derived factors than those from the added model (Figs. 3B and S3B). This indicates that summation partially entangles modality-specific signals, whereas concatenation preserves them as separable latent dimensions. These findings are likewise reflected on UMAP embeddings of the four modalities where concatenation yields the best-resolved substructure (Fig. S4). Together, these results explain why concatenating spliced and unspliced features supports higher revealable rank—by retaining complementary structure that is obscured when the two modalities are collapsed.

### 2.4 Concat-specific factors are driven by modality-skewed gene weights

To examine why specific concatenated factors are lost in the added representation, we plotted spliced versus unspliced loading weights for genes within concat-specific factors from the PBMC dataset (Fig. 4). Rather than clustering near the diagonal—which would indicate redundant contributions—high-loading genes were strongly displaced toward one axis. In unspliced-dominant factors (e.g. Factors 23 and 28), key genes loaded primarily through unspliced counts, consistent with nascent transcriptional programs; in spliced-dominant factors (Factors 34 and 35), loading came primarily from spliced counts, consistent with stable transcript abundance. Summing the modalities erases this directional information, reducing factor separability.

**Figure 4:**
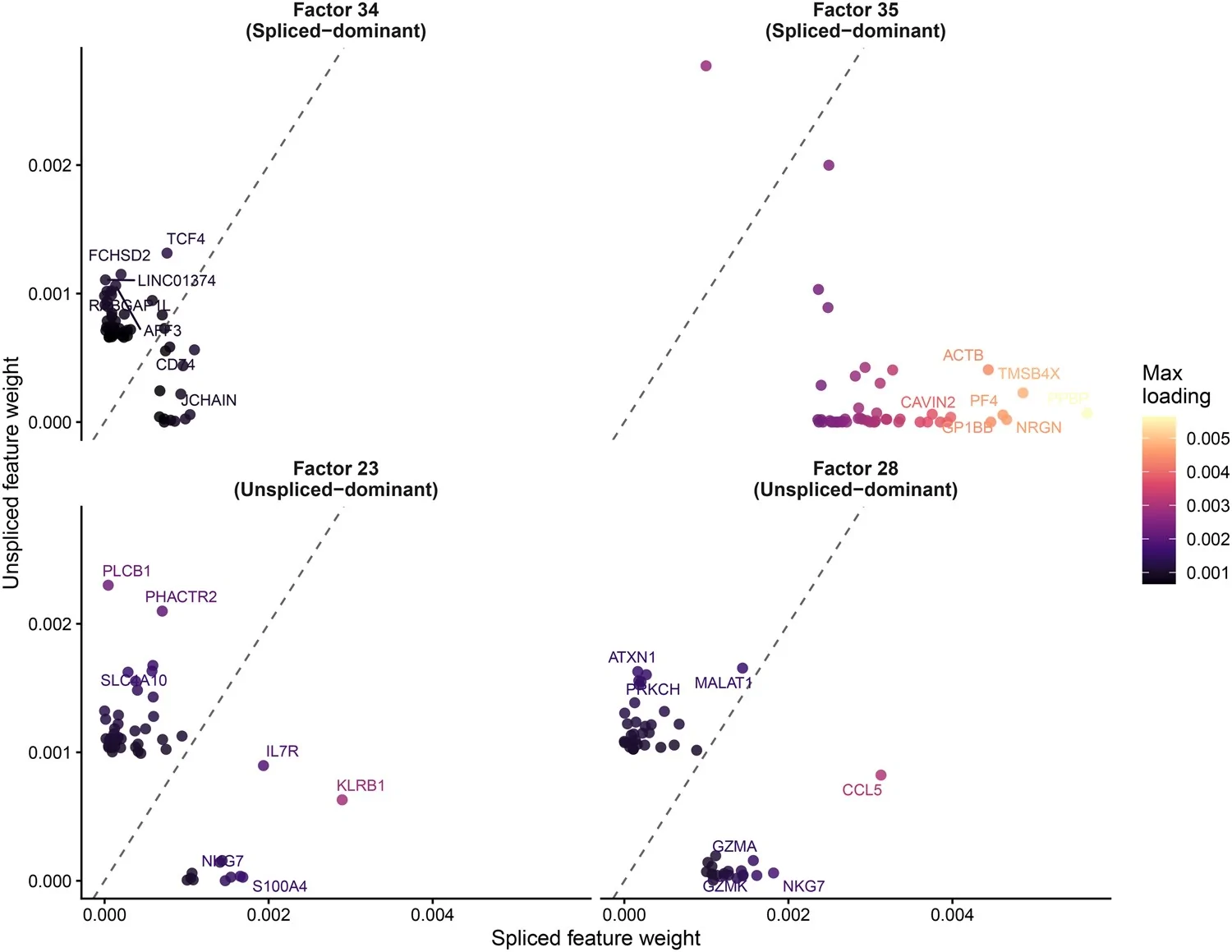
Concat-specific factors are driven by genes with strongly asymmetric modality contributions, explaining why summation obscures latent programs recoverable only in the concatenated representation. For selected concatenated NMF factors from the PBMC dataset, each point represents a gene plotted by its spliced (x-axis) and unspliced (y-axis) loading weight within the same factor. The dashed diagonal indicates equal contribution from both modalities. Genes in unspliced-dominant factors (e.g. Factors 23 and 28) load primarily through unspliced counts, consistent with nascent transcriptional programs; genes in spliced-dominant factors (e.g. Factors 34 and 35) load primarily through spliced counts, consistent with stable transcript abundance. Collapsing modalities by summation erases this directional information, reducing the separability of factors recoverable only in the concatenated representation.

### 2.5 Added model factors preferentially match modality-specific concatenated factors

To characterize how modality-specific structure is represented in the added model, we compared each added model factor to both the spliced-dominant and unspliced-dominant factors of the concatenated model using cosine similarity. Of 36 added model factors, 22 matched unspliced-dominant concat factors more closely than spliced-dominant factors, while 14 showed the reverse preference. Added factors that preferentially matched unspliced concat factors corresponded to genuinely unspliced-dominant latent programs (median unspliced fraction 0.63), whereas those matching spliced concat factors corresponded to spliced-dominant programs (median unspliced fraction 0.41; Wilcoxon rank-sum test, *P* = 7.5 × 10^−5^). This demonstrates that the added model retains partial modality-specific structure but conflates spliced and unspliced programs within individual factors rather than resolving them as separable latent dimensions, providing a mechanistic explanation for why the added representation supports lower revealable rank than the concatenated representation.

### 2.6 Modality-specific NMF factors correspond to distinct biological programs

To extend this analysis beyond selected concat-specific examples, we examined the relative contribution of each modality to all latent factors and evaluated their functional enrichment. For each factor in the concatenated model, we quantified the proportion of total feature loadings attributable to spliced versus unspliced transcripts and performed Gene Set Enrichment Analysis (GSEA) independently on each modality.

We used MSigDB Gene Ontology (C5) terms for GSEA, subsetting MSigDB pathways to the gene universe present in the single-cell transcriptomics data, and vice versa. Many latent factors had a preference for one modality over the other, indicating that NMF assigns distinct transcriptional signals to separable dimensions (Fig. 5A).

**Figure 5:**
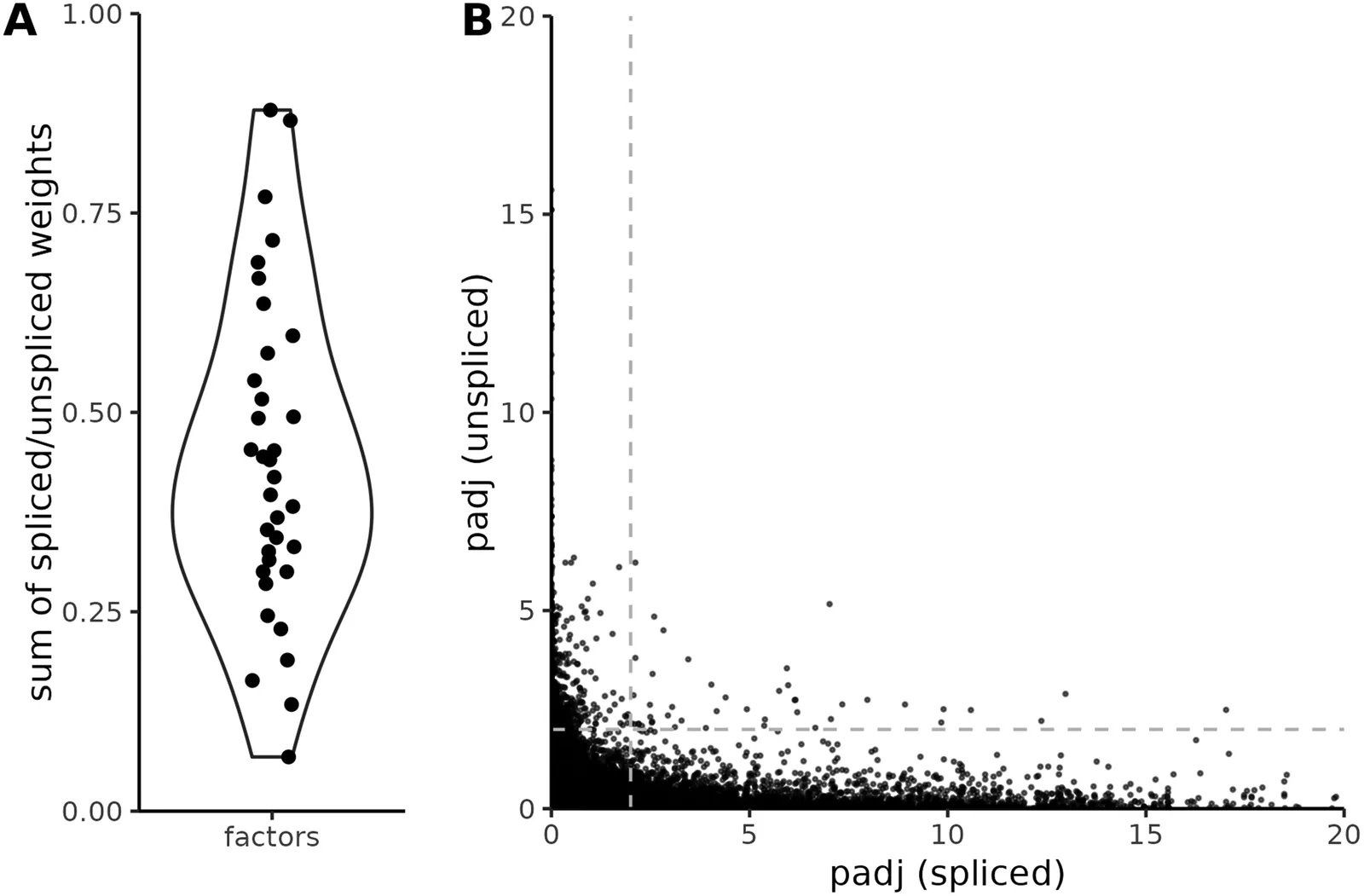
Spliced and unspliced features encode biologically distinct transcriptional programs, not redundant ones. (A) Most latent factors have a clear modality preference—loading predominantly on spliced or unspliced features—indicating that NMF assigns transcriptional signals to separable dimensions rather than mixing them. (B) GSEA *P* values derived independently from spliced and unspliced loadings within the same factor show no correlation, demonstrating that the two modalities capture distinct biological processes even within shared latent dimensions.

Consistent with this factor-specific modality preference, biological enrichment patterns differed markedly between spliced and unspliced features within the same factor. Across factors, BH-adjusted GSEA significance derived separately from spliced versus unspliced gene weights showed no appreciable association (Fig. 5B), consistent with the two modalities emphasizing largely distinct regulatory programs within shared latent dimensions. Because enrichment was computed against modality-specific gene universes and significance reflects both effect size and the differing statistical power of the sparser unspliced layer, we interpret this pattern as evidence of non-redundancy rather than as a quantitative measure of program divergence. Together, these results show that preserving spliced and unspliced features yields latent factors that are not only structurally distinct but also biologically interpretable, providing a functional explanation for the increased revealable rank observed when the modalities are modeled jointly.

### 2.7 Concatenated latent factors exhibit regulatory complementarity between spliced and unspliced signals

In addition to factors dominated by a single modality, the concatenated model learned latent dimensions in which spliced and unspliced features contributed substantially within the same factor. To assess whether these shared factors reflected coherent biological programs, we performed GSEA separately on spliced and unspliced loadings for each factor. Across multiple factors, spliced and unspliced features within the same latent dimension highlighted distinct biological processes, indicating complementary regulatory signatures captured within a single factor (Fig. 6).

**Figure 6:**
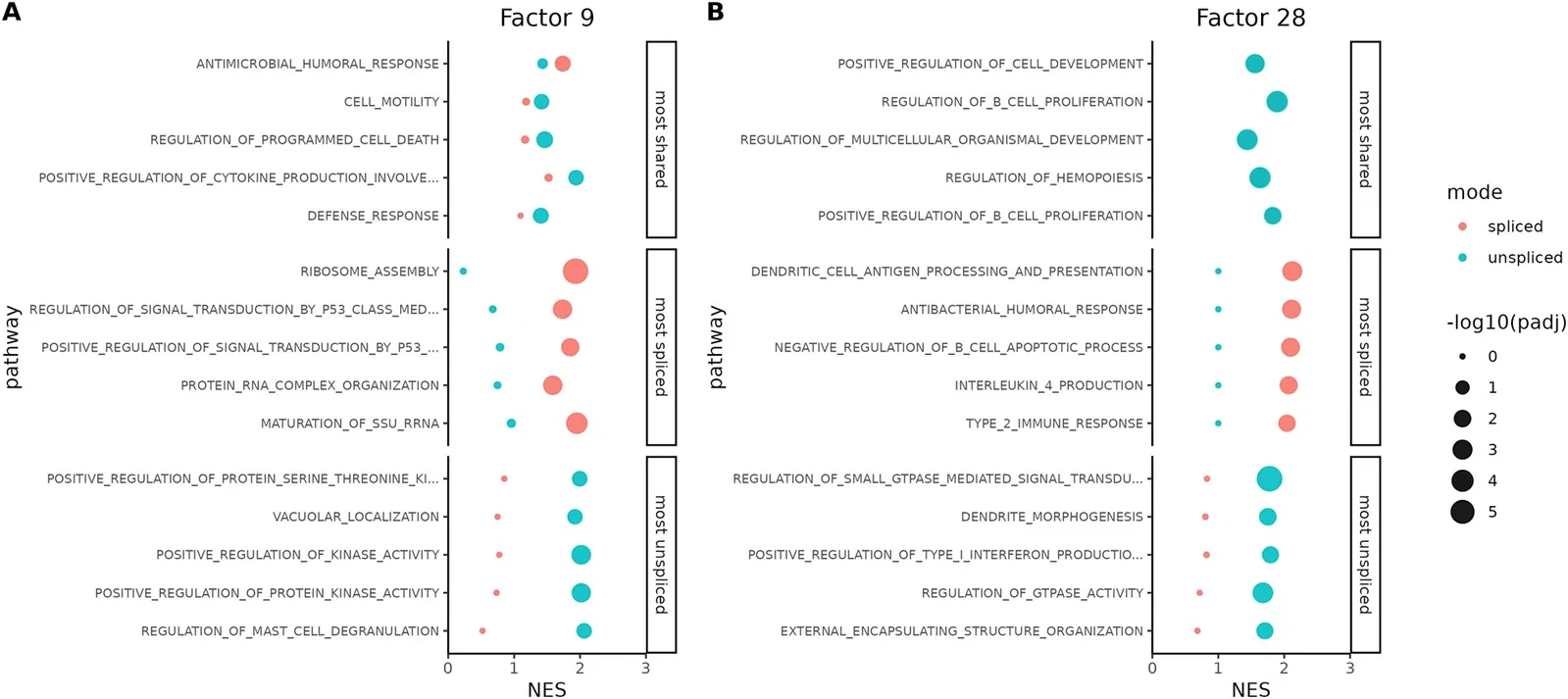
Concatenated latent factors encode complementary regulatory signatures across spliced and unspliced signals. Gene Set Enrichment Analysis (GSEA) performed separately on spliced and unspliced feature loadings within selected latent factors from the concatenated model. (A) Factor 9 from the heart dataset shows unspliced enrichment for signaling and mast-cell–associated pathways, whereas spliced enrichment emphasizes ribosome assembly and p53-related processes. (B) Factor 28, also from the heart dataset, exhibits a similar separation, with unspliced features enriched for morphogenesis- and future-state programs and spliced features reflecting immune activation.

For example, in Factor 9 of the heart dataset, unspliced loadings were enriched for kinase signaling and mast-cell degranulation, whereas spliced loadings emphasized ribosome assembly and p53 signaling. Similarly, in Factor 28, unspliced enrichments captured programs associated with morphogenesis and cell-state specification, while spliced enrichments reflected immune activation and translational activity. These patterns generalized across factors (more examples presented in Fig. S5), with unspliced features frequently enriched for transcriptional activation, signaling, and developmental pathways, whereas spliced features preferentially captured translation, oxidative metabolism, cell-cycle regulation, and other homeostatic processes.

Together, these results show that concatenating spliced and unspliced transcripts enables a single latent factor to encode multiple, functionally distinct aspects of a biological program, without requiring explicit kinetic or temporal modeling. By preserving both modalities, NMF captures complementary regulatory signatures that are otherwise conflated when transcript origin is ignored.

### 2.8 Concatenated representation recovers more unique biological programs than added representation

To test whether higher revealable rank translates to broader biological discovery, we counted gene sets significant (*P*adj < .05) in one model but absent from the other across all four datasets (Table 2). Concat recovered substantially more exclusive terms across datasets, representing programs entirely absent from the added model rather than merely attenuated. Critically, this gain is not a simple consequence of concat having more factors. The ratio of exclusive terms (Brain 2.0×, Heart 1.2×, Neuron 1.1×) exceeds the corresponding ratio of revealable ranks (Brain 1.08×, Heart 1.15×, Neuron 1.07×) in every dataset, and most dramatically in Brain, indicating that the additional concatenated factors recover disproportionately more unique biology per factor than a factor-count explanation would predict. In PBMC, where the rank difference between concat and added was smallest (44.7 versus 42.0), exclusive term recovery was comparable (Concat: 530, Added: 556), consistent with biological program gain tracking the magnitude of representational capacity increase rather than factor count alone.

**Table 2:** Gene sets significant (*P*adj < .05) in one model but absent from the other across all factors, counted from a single representative replicate per condition at the dataset-specific revealable rank.

Dataset	Concat exclusive	Added exclusive
**Brain**	922	457
**Heart**	865	728
**Neuron**	695	629
**PBMC**	530	556

The advantage of concat exceeds what its modest rank increase alone would predict—exclusive term ratios consistently outpace rank ratios across datasets—indicating that biological program recovery tracks resolvable modality-specific structure rather than factor count.

## 3 Discussion

A central result of this study is that preserving splicing status in single-cell RNA-seq data increases the amount of latent structure that can be stably recovered. By treating spliced and unspliced transcripts as independent features, rather than collapsing them into a single expression matrix, we show that common single-cell datasets support higher revealable rank under cross-validated NMF, with the magnitude of the advantage varying by dataset. This increase reflects genuine, explainable structure rather than overfitting, indicating that standard preprocessing conventions discard resolvable information before analysis begins.

Our findings clarify that spliced and unspliced counts encode complementary, not redundant, signals. Across datasets, factors learned from spliced and unspliced layers exhibited minimal overlap, while concatenated models preserved modality-specific structure more faithfully than additive representations. These results establish that summation obscures distinctions that low-rank models can otherwise resolve when modalities are presented jointly. From a representation perspective, splicing status functions as an informative axis of variation rather than a nuisance variable.

Evidence from other domains likewise indicates that ablating individual views degrades downstream performance in multi-view models (Xiong *et al*. 2025), supporting the general principle—applied here to transcript modality—that preserving complementary views can improve recovery of meaningful structure.

Importantly, the additional structure exposed by concatenation is biologically interpretable. Factors dominated by spliced or unspliced features preferentially enriched for distinct gene programs, and even within shared factors, the two modalities highlighted different aspects of the same biological processes. These patterns are consistent with unspliced transcripts reflecting transcriptional activation and nascent responses, while spliced transcripts emphasize established or steady-state programs.

Without invoking kinetic assumptions, this dual representation provides a richer and more nuanced view of cellular state. As a concrete illustration of a question that summation cannot answer, consider Factor 9 of the Heart dataset: its unspliced loadings enrich for kinase signaling and mast-cell degranulation while its spliced loadings enrich for ribosome assembly and p53 signaling (Fig. 6). Asking whether a given cell population is actively engaging a signaling response versus maintaining a steady-state translational program is answerable only when transcript origin is preserved—in the added representation these two processes collapse into a single factor and become indistinguishable. The exclusive-term analysis (Table 2) generalizes this point: hundreds of GO programs per dataset are recoverable only when splicing status is retained, defining a concrete class of transcriptional-state questions that the conventional summed matrix cannot address.

Methodologically, we introduce revealable rank as a practical, cross-validated measure of information content that enables principled comparison across feature representations. While our analysis focuses on NMF, the core insight is representation-centric rather than model-specific: preserving splicing status expands the expressible subspace available to downstream models. This aligns with transcript-resolved work showing that gene-level summaries can mask isoform-level variation (Chen *et al*. 2026), and multiview studies showing that complementary views improve structure recovery (Xiong *et al*. 2025).

These findings carry immediate implications for large-scale single-cell atlases. As community resources increasingly standardize preprocessing and archive expression matrices for reuse, decisions about whether to collapse or preserve splicing layers directly affect the biological structure that future analyses can uncover. Retaining spliced and unspliced counts as first-class features substantially increases analytical flexibility, interpretability, and downstream reuse potential.

Our framework preserves the distinction between spliced and unspliced transcript abundance, which is conceptually separate from isoform-resolved alternative splicing. The two are complementary: spliced/unspliced status captures transcriptional timing and processing state, whereas alternative splicing determines which mature isoform is produced—a distinction with direct functional consequences, as in the *VEGFA* locus, where pro- and anti-angiogenic isoforms arising from alternative splice-site selection exert opposing biological effects (Mamer *et al*. 2020, White and Bix 2023, Zheng *et al*. 2024). A natural extension of the present approach is to treat isoform-, exon-, and junction-level quantities as additional first-class feature blocks within the same concatenation strategy. Transcript-isoform abundance, exon inclusion/exclusion metrics, splice-junction usage, velocity-informed latent variables, and multimodal regulatory priors could each be appended as modality-specific blocks, allowing NMF or related factorizations to resolve isoform-level programs alongside the spliced/unspliced axis studied here. We frame the current work as a foundational modality-preservation result rather than a complete isoform-level splicing pipeline.

We selected NMF deliberately rather than a nonlinear latent-variable model. Because the central question is representational—whether preserving splicing status expands the recoverable subspace—an interpretable, additive factorization lets us attribute recovered structure directly to spliced versus unspliced features and inspect modality contributions per factor, which is not straightforward in variational autoencoders, contrastive frameworks, or other multimodal deep architectures. The core insight is model-agnostic: preserving splicing status enlarges the expressible feature space available to any downstream model. We therefore expect the benefit to transfer to nonlinear architectures, where spliced and unspliced blocks could enter as separate modality channels, and we identify systematic comparison against such methods as a direction for future work.

The complementarity we observe also has implications beyond descriptive factorization. Because unspliced loadings preferentially capture nascent, transcriptionally active programs while spliced loadings emphasize steady-state output, preserving both modalities retains information directly relevant to transcriptional dynamics. This structure could in principle improve RNA-velocity estimation, transcriptional-state forecasting, regulatory-network inference, and trajectory modeling by supplying explicitly separated dynamic and steady-state signals as inputs, rather than requiring downstream models to disentangle them from a summed matrix. Evaluating whether modality preservation measurably improves these predictive tasks is an important next step.

Several limitations bound the present conclusions. First, we estimated revealable rank under a single, uniform entry-wise masking scheme, chosen so that differences across representations reflect the representations themselves rather than validation design. Alternative schemes—cell-wise, gene-wise, or block-wise masking—emphasize different aspects of latent recovery, and although all representations here were evaluated identically (making the comparison internally consistent), the absolute robustness of $k^{*}$ to masking strategy remains to be characterized. Second, all representations share a common preprocessing pipeline; because per-cell log-normalization operates over a feature set that differs in size between the added and concatenated inputs, we cannot fully exclude a normalization contribution to the observed differences, and systematic evaluation of feature selection, variance stabilization, and modality reweighting is warranted. Third, our biological validation is enrichment-based; we have not benchmarked whether higher revealable rank improves supervised downstream tasks such as clustering, cell-type classification, or trajectory inference (e.g. via ARI, NMI, silhouette, or classifier accuracy on well-annotated references), which we regard as the priority next step. Finally, the advantage of preserving splicing status is not universal: it is expected to be smallest when signal is dominated by stable terminal cell identity, when transcriptional dynamics are weak, when sequencing depth is limited, or when the unspliced layer is sparse or noisy—conditions under which added and concatenated representations may perform comparably, as the modest PBMC advantage illustrates.

In summary, splicing status is not merely auxiliary metadata for dynamic modeling, it is a foundational component of transcriptional signal. Preserving spliced and unspliced layers expands the dimensionality of discoverable biology without altering experimental design or requiring new assays. For atlas builders, method developers, and curators of public single-cell resources, this work argues for a simple but consequential shift in practice: store more of what the data already contain, and let downstream models decide what structure can be revealed.

## 4 Methods

### 4.1 Single-cell RNA-seq datasets

We analysed four 10x Genomics scRNA-seq datasets (PBMC, heart, brain, neuron) considered in Gorin *et al*. (2022). In their study, datasets were obtained from 10× Genomics and processed with standard preprocessing workflows to quantify spliced and unspliced molecules. Gorin *et al.* made available scripts and loom files generated by these workflows, which we used. In this work, we did not reprocess raw sequencing data or regenerate spliced/unspliced counts; instead, we used the released count matrices exactly as provided. Loom containers were parsed in R via reticulate, and spliced and unspliced layers were exported to .npz format before being cast to sparse dgCMatrix objects using the Matrix package. Cells were retained if they contained between 200 and 15 000 detected features and between 500 and 75 000 total UMIs. Genes detected in fewer than 10 cells were removed. No additional feature selection or variance filtering was applied.

### 4.2 Construction of spliced and unspliced feature matrices

From the raw spliced and unspliced count matrices, we constructed four modality-specific input matrices:

1. Spliced-only (*s*).
2. Unspliced-only (*u*).
3. Added (s+u): per-gene alignment by gene symbol, followed by element-wise summation of the raw counts.
4. Concatenated (s⊕u): row-wise concatenation of the raw spliced and unspliced counts, preserving gene identity by appending modality-specific suffixes (_s, _us).

Gene sets were identical between spliced and unspliced matrices. No reweighting was applied between modalities, and no group-wise or batch normalization was performed. All four representations were analysed in parallel in downstream experiments.

### 4.3 Preprocessing and normalization

All input matrices were normalized using Seurat standard log-normalization with default parameters. No additional feature selection or variance filtering was applied for NMF. For PCA analyses, 2000 highly variable genes were selected and gene-wise centering and unit-variance scaling was applied using the standard Seurat workflow.

### 4.4 Non-negative matrix factorization

For each dataset and each modality construction (*s*, *u*, s+u, s ⊕ u), we applied Non-negative Matrix Factorization (NMF) to decompose the normalized expression matrix $X\in R_{\geq 0}^{p\times n}$ into a low-rank product


$$X\approx W^{⊤}H,$$

where *p* denotes the number of features, *n* the number of cells, and *k* the factorization rank. The matrix $W\in R_{\geq 0}^{k\times p}$ contains non-negative gene loadings for each factor, and $H\in R_{\geq 0}^{k\times n}$ contains the corresponding non-negative cell loadings. We evaluated ranks k∈{2,3,…,80} for all datasets and modalities.

Models were fit by minimizing squared reconstruction error under non-negativity constraints,


$$\underset{W\geq 0,\;H\geq 0}{\min}\|X-W^{⊤}H{\|}_{F}^{2},$$

using an alternating least squares scheme with non-negative least squares updates. All factorization was performed using our open-source singlet implementation (C++ with R bindings), which is optimized for sparse inputs and parallel execution.

For each replicate, factor matrices were initialized from a uniform distribution on (0,1) and trained for a fixed number of epochs (50), independent of dataset, modality, or rank. Training curves confirmed that held-out error plateaued well within 50 epochs at ranks at or below the estimated revealable rank across all datasets and representations (Fig. S2). At substantially higher ranks, particularly for the Unspliced-only representation in PBMC, models exhibited continued slow improvement at epoch 50, indicating incomplete convergence in the overfit regime; however, since $k^{*}$ selection relies only on the stable-region behavior, this does not affect the reported revealable rank estimates. The equal epoch budget applies uniformly to the added (*p*-feature) and concatenated (2*p*-feature) inputs, ensuring that representations are compared at matched optimization effort. No regularization, modality reweighting, or other stopping criteria were applied.

### 4.5 Rank selection via cross-validation

To assess generalization performance and determine the effective dimensionality supported by each data representation, we employed an entry-wise cross-validation strategy based on sparse holdout masking, as implemented in the cross_validate_nmf function in the singlet R package. For each dataset and modality, a fixed subset of matrix entries was designated as a test set. Each entry (i,j) was assigned to the held-out set with probability approximately 0.05, using a deterministic two-dimensional congruential pseudorandom number generator indexed by (i,j) and a replicate-specific seed. Masking was applied uniformly over all entries, including both zero and nonzero values.

Let Ω denote the set of held-out indices and $\Omega^{c}$ its complement. During model fitting, NMF updates were performed using only entries in $\Omega^{c}$, while performance was evaluated on Ω. The same mask was reused across all epochs for a given rank, and for all ranks in each replicate, ensuring that cross-validation comparisons reflected model capacity across ranks rather than stochastic resampling effects.

Model performance was quantified using mean squared reconstruction error on the held-out entries,


$${\text{MSE}}_{\Omega }=\frac{1}{\left|\Omega \right|}\sum_{(i,j)\in \Omega }{\left(X_{ij}-{\hat{X}}_{ij}\right)}^{2},$$

where $\hat{X}=W^{⊤}H$ denotes the reconstructed matrix.

For each dataset, modality, and rank, we fit three independent replicates distinguished by random seeds. The same set of seeds was reused across ranks to ensure stable cross-rank comparisons. Cross-validation curves and downstream summaries report the mean held-out error across replicates.

### 4.6 Determination of revealable rank

For each dataset, representation, and replicate, we fit NMF across a contiguous span of ranks and tracked the held-out reconstruction error ${\text{MSE}}_{\Omega }(k)$ after convergence. For each replicate, $k^{*}$ was selected as the largest rank within the near-minimum plateau of the cross-validation curve, defined as the contiguous region of ranks achieving held-out reconstruction error within the range of minimum values observed across the rank sweep, immediately preceding the onset of sustained overfitting (Tunnell *et al*. 2025). This criterion selects the most expressive stable model rather than the conservative global minimum, which can underestimate the true stable rank due to initialization-dependent variation within the plateau region. Three independent replicates were run per dataset and representation using distinct random seeds. Reported $k^{*}$ values in Table 1 are the mean ± standard deviation of the three replicate-specific selections.

### 4.7 Factor similarity and cross-modality matching

To quantify correspondence between latent factors learned from different input modalities, we compared NMF gene-loading matrices across models. For each modality-specific model, we formed a factor-by-factor similarity matrix *S* between columns of the two gene-loading matrices (e.g. using dot products of $\ell_{2}$-normalized loading vectors, which is equivalent to cosine similarity). We then computed an optimal one-to-one correspondence between factors by solving a bipartite matching problem using RcppML::bipartiteMatch. Specifically, factors were matched to maximize total similarity (or equivalently to minimize a cost matrix derived from similarity, e.g. C=1−S). All matching was performed on factors learned at the revealable rank for each dataset and modality.

### 4.8 Added model factor matching analysis

Cosine similarity was computed between each added model factor and all concatenated model factors, separately for spliced-dominant and unspliced-dominant concat factors ($\ell_{2}$-normalized loading vectors; dominance threshold: 50% unspliced feature weight). Each added factor was classified by which maximum similarity was greater; group differences in unspliced fraction were assessed by Wilcoxon rank-sum test.

### 4.9 Gene set enrichment analysis

To interpret the biological programs captured by individual NMF factors, we performed Gene Set Enrichment Analysis (GSEA) on factor-specific gene loadings. For each factor, genes were ranked by their normalized loading weights in the gene-loading matrix *W*. In the concatenated modality, spliced and unspliced features were analysed separately: GSEA was performed independently on the spliced-derived gene weights and the unspliced-derived gene weights associated with the same latent factor. Enrichment analysis was conducted using the fgsea package in R, with gene sets drawn from the MSigDB Gene Ontology C5 collection. The gene universe for each analysis was restricted to genes present in the corresponding spliced or unspliced layer after quality control. This strategy enables direct comparison of biological processes emphasized by spliced versus unspliced contributions within shared latent factors, facilitating interpretation of modality-specific and temporally complementary regulatory programs.

### 4.10 Exclusive gene set analysis

Gene sets (MSigDB GO C5) significant in the concatenated model (*P*adj < .05) but absent from the added model were counted, and vice versa, across all four datasets. A gene set was considered exclusive if it met the threshold in any factor of one model and no factor of the other. For each dataset, exclusive terms were counted from the single replicate whose revealable rank was closest to the modality-specific mean reported in Table 1.

### 4.11 Software and data availability

All datasets analysed in this study are publicly available from the Caltech Data repository (Gorin and Chari 2022). DOI: 10.22002/D1.20030. All NMF analyses were performed using the singlet library, which provides a C++ implementation of non-negative matrix factorization with an R interface. The source code for singlet is publicly available at https://github.com/zdebruine/singlet, including the cross_validate_nmf function used in this study. Complete scripts required to reproduce all figures, cross-validation analyses, and downstream results presented in this manuscript are available at https://github.com/Arya-86/nmf-spliced-unspliced. All analyses were executed on CPU-only systems with multi-threaded execution (up to 128 threads); results were invariant to thread count in our implementation and do not necessarily require HPC resources due to the memory and computational efficiency of the singlet C++ implementation and relatively small size of these datasets. All random number generation was controlled via fixed seeds, and identical seeds were reused across ranks within each replicate to ensure deterministic and comparable cross-validation results. Given identical inputs, seeds, and software versions, all reported results are fully reproducible.

## Supplementary Material

vbag263_Supplementary_Data

## Data Availability

The datasets analysed in this study are publicly available from CaltechDATA (Gorin and Chari 2022) at https://doi.org/10.22002/D1.20030. The singlet R package is available at https://github.com/zdebruine/singlet, and code to reproduce all analyses and figures is available at https://github.com/Arya-86/nmf-spliced-unspliced.
