# Supplementary material for: Joint representation of spliced and unspliced RNA expands information content in single-cell expression data
Source: Bioinform Adv. 2026 Oct 22;6(1):vbag263. doi: 10.1093/bioadv/vbag263 (PMC13718436; doi:10.1093/bioadv/vbag263)
Supplement: Supplementary file 1 [file vbag263_supplementary_data.zip › S1C_neuron_pca.pdf]

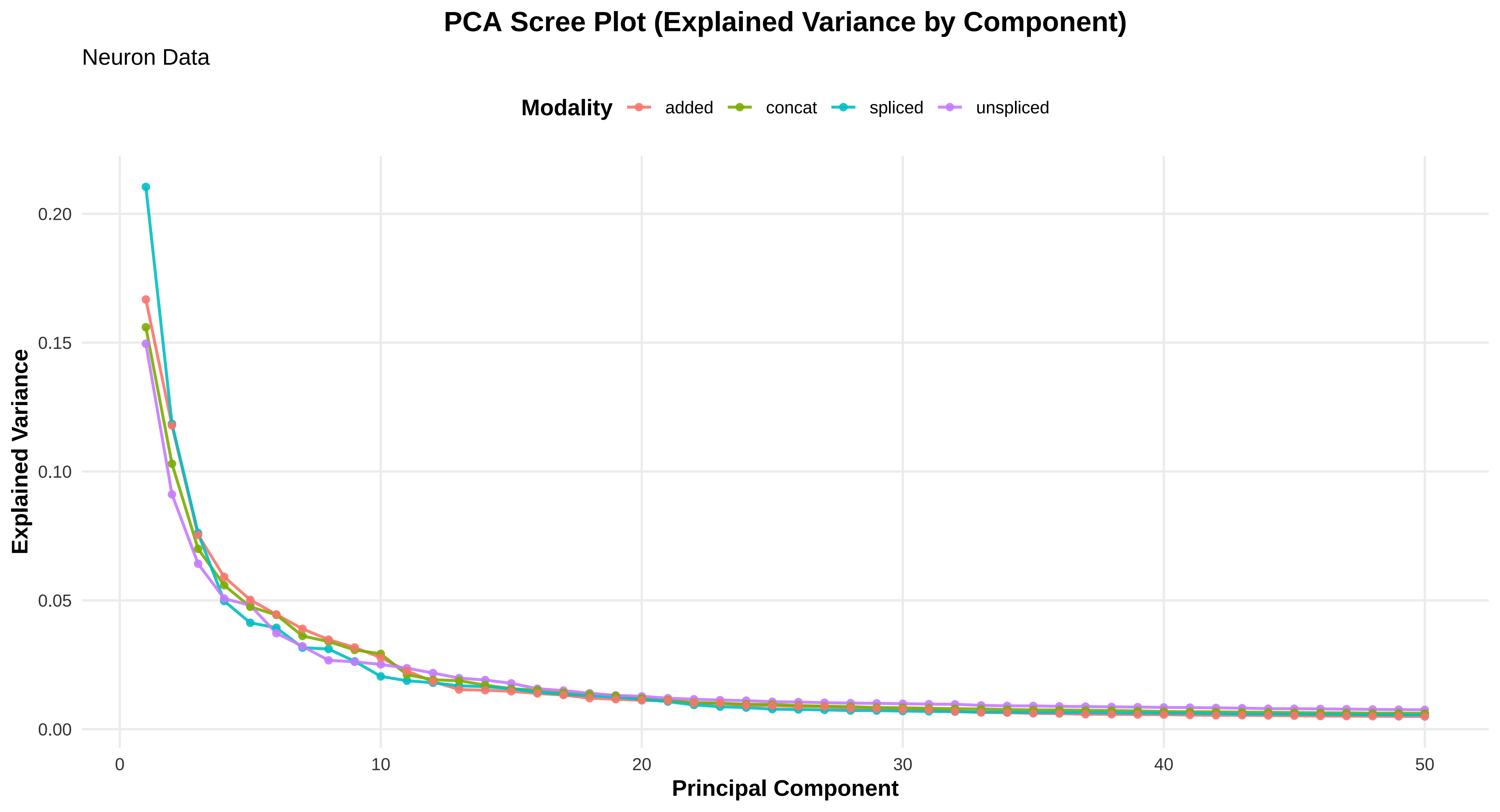
PCA Scree Plot (Explained Variance by Component)
Neuron Data
Modality
added
concat
spliced
unspliced
Explained Variance
0.20
0.15
0.10
0.05
0.00
Principal Component
0
10
20
30
40
50
