# Supplementary material for: Joint representation of spliced and unspliced RNA expands information content in single-cell expression data
Source: Bioinform Adv. 2026 Oct 22;6(1):vbag263. doi: 10.1093/bioadv/vbag263 (PMC13718436; doi:10.1093/bioadv/vbag263)
Supplement: Supplementary file 1 [file vbag263_supplementary_data.zip › S2A_pbmc_cv.pdf]

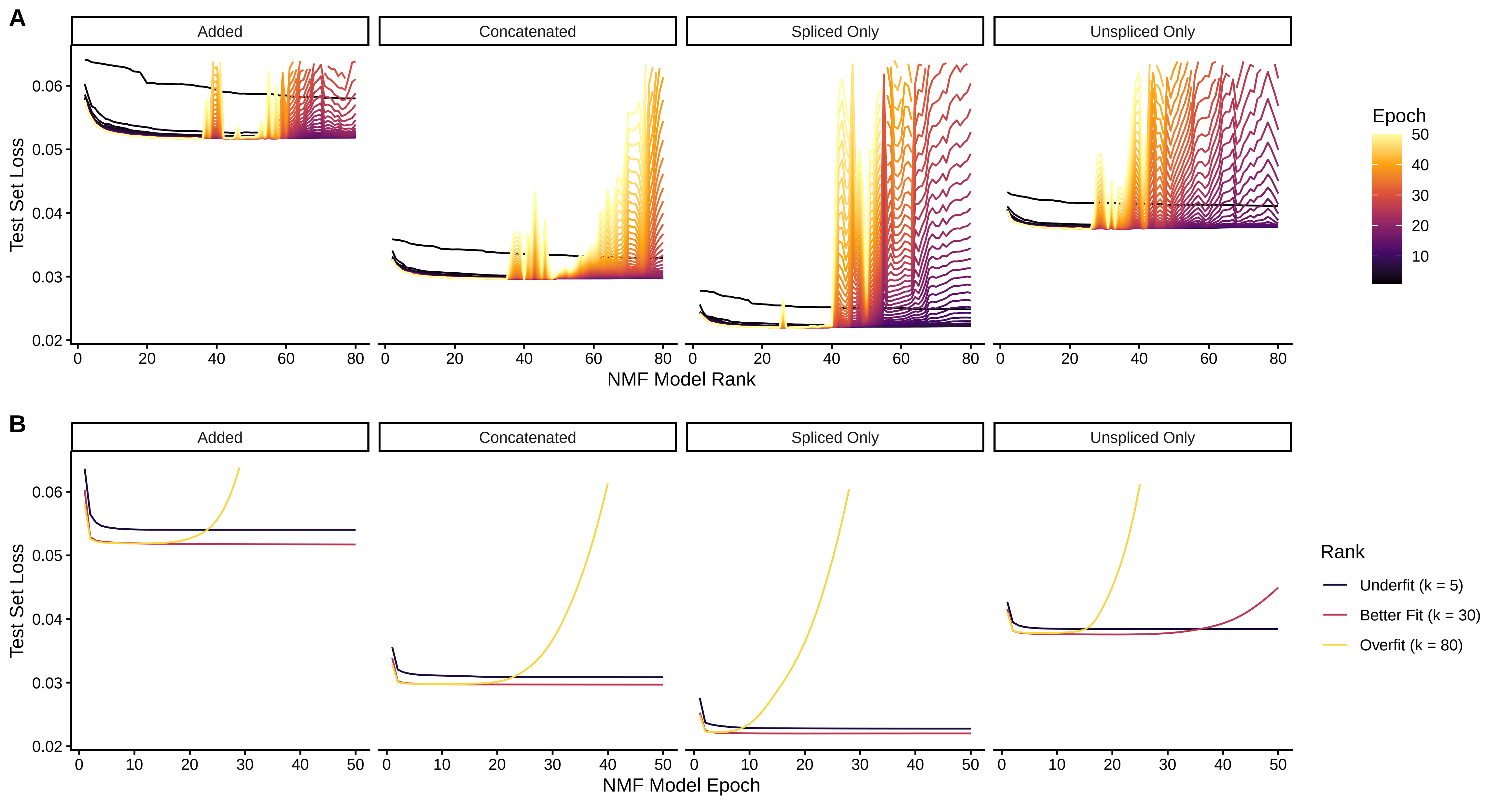

A
Added
Concatenated
Spliced Only
Unspliced Only
Test Set Loss
0.06
0.05
0.04
0.03
0.02
0
20
40
60
80
NMF Model Rank
Epoch
50
40
30
20
10
B
Added
Concatenated
Spliced Only
Unspliced Only
Test Set Loss
0
10
20
30
40
50
NMF Model Epoch
Rank
Underfit (k = 5)
Better Fit (k = 30)
Overfit (k = 80)
