# Supplementary material for: Joint representation of spliced and unspliced RNA expands information content in single-cell expression data
Source: Bioinform Adv. 2026 Oct 22;6(1):vbag263. doi: 10.1093/bioadv/vbag263 (PMC13718436; doi:10.1093/bioadv/vbag263)
Supplement: Supplementary file 1 [file vbag263_supplementary_data.zip › S3.pdf]

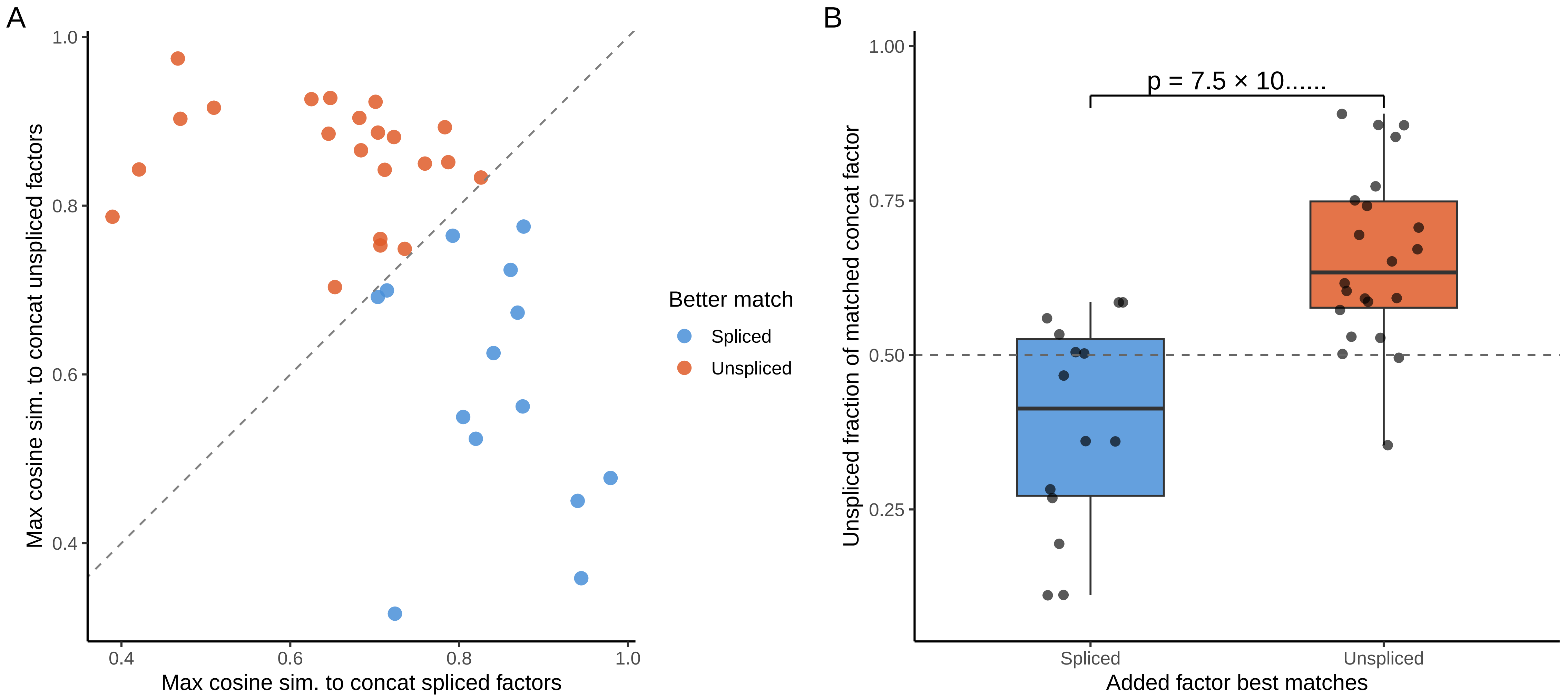

A
B
Max cosine sim. to concat unspliced factors
Max cosine sim. to concat spliced factors
1.0
0.8
0.6
0.4
Better match
Spliced
Unspliced
p = 7.5 × 10......
Unspliced fraction of matched concat factor
1.00
0.75
0.50
0.25
Added factor best matches
