# Supplementary material for: Joint representation of spliced and unspliced RNA expands information content in single-cell expression data
Source: Bioinform Adv. 2026 Oct 22;6(1):vbag263. doi: 10.1093/bioadv/vbag263 (PMC13718436; doi:10.1093/bioadv/vbag263)
Supplement: Supplementary file 1 [file vbag263_supplementary_data.zip › S5_GSEA_additional_factors.pdf]

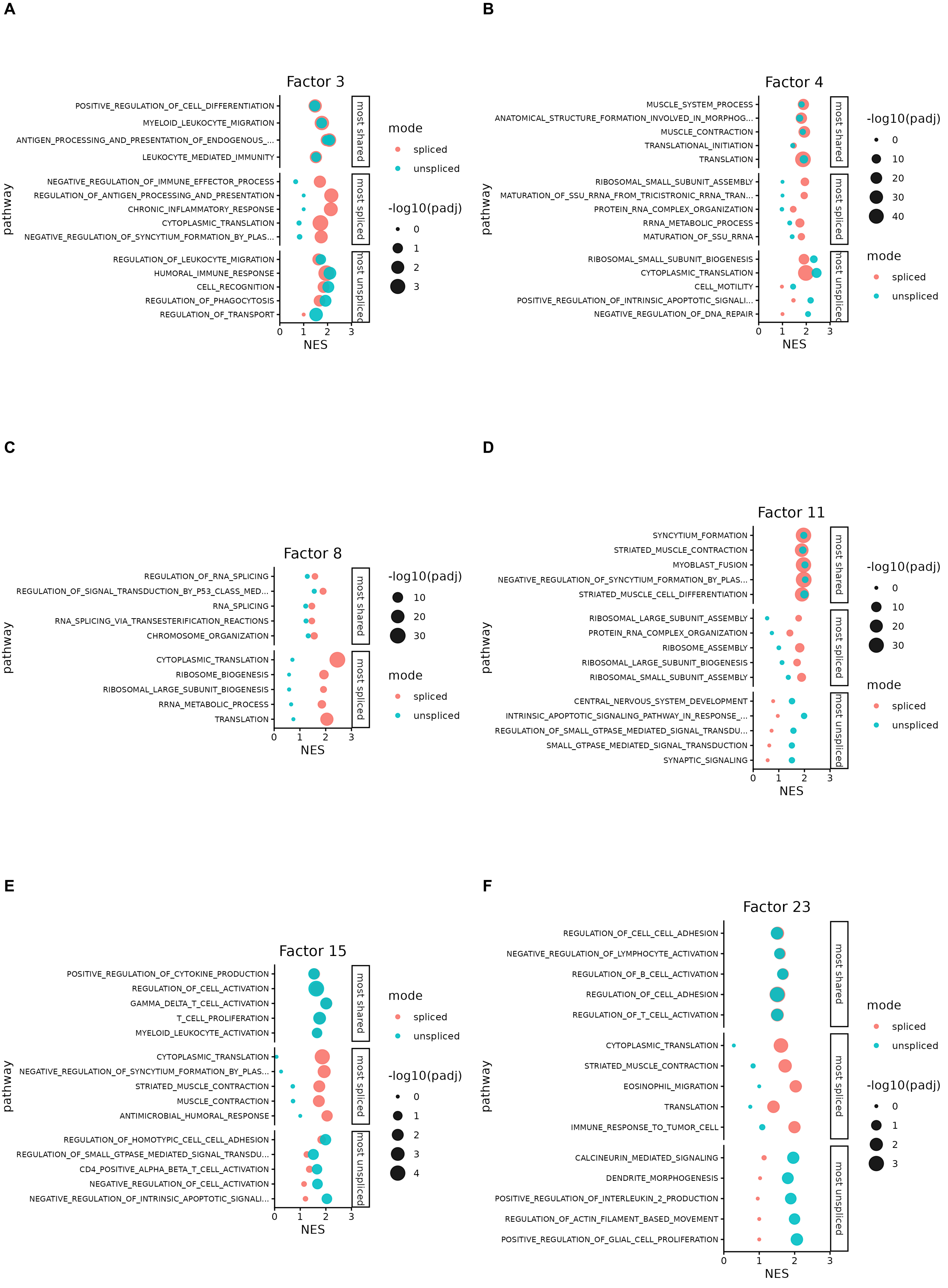
A
Factor 3
POSITIVE_REGULATION_OF_CELL_DIFFERENTIATION
MYELOID_LEUKOCYTE_MIGRATION
ANTIGEN_PROCESSING_AND_PRESENTATION_OF_ENDOGENOUS_...
LEUKOCYTE_MEDIATED_IMMUNITY
NEGATIVE_REGULATION_OF_IMMUNE_EFFECTOR_PROCESS
REGULATION_OF_ANTIGEN_PROCESSING_AND_PRESENTATION
CHRONIC_INFLAMMATORY_RESPONSE
CYTOPLASMIC_TRANSLATION
NEGATIVE_REGULATION_OF_SYNCYTIUM_FORMATION_BY_PLAS...
REGULATION_OF_LEUKOCYTE_MIGRATION
HUMORAL_IMMUNE_RESPONSE
CELL_RECOGNITION
REGULATION_OF_PHAGOCYTOSIS
REGULATION_OF_TRANSPORT
most shared
most spliced
most unspliced
pathway
NES
0 1 2 3
mode
spliced
unspliced
-log10(padj)
0 1 2 3
B
Factor 4
MUSCLE_SYSTEM_PROCESS
ANATOMICAL_STRUCTURE_FORMATION_INVOLVED_IN_MORPHOG...
MUSCLE_CONTRACTION
TRANSLATIONAL_INITIATION
TRANSLATION
RIBOSOMAL_SMALL_SUBUNIT_ASSEMBLY
MATURATION_OF_SSU_RRNA_FROM_TRICISTRONIC_RRNA_TRAN...
PROTEIN_RNA_COMPLEX_ORGANIZATION
RRNA_METABOLIC_PROCESS
MATURATION_OF_SSU_RRNA
RIBOSOMAL_SMALL_SUBUNIT_BIOGENESIS
CYTOPLASMIC_TRANSLATION
CELL_MOTILITY
POSITIVE_REGULATION_OF_INTRINSIC_APOPTOTIC_SIGNALI...
NEGATIVE_REGULATION_OF_DNA_REPAIR
most shared
most spliced
most unspliced
pathway
NES
0 1 2 3
-log10(padj)
0 10 20 30 40
mode
spliced
unspliced
C
Factor 8
REGULATION_OF_RNA_SPLICING
REGULATION_OF_SIGNAL_TRANSDUCTION_BY_P53_CLASS_MED...
RNA_SPLICING
RNA_SPLICING_VIA_TRANSESTERIFICATION_REACTIONS
CHROMOSOME_ORGANIZATION
CYTOPLASMIC_TRANSLATION
RIBOSOME_BIOGENESIS
RIBOSOMAL_LARGE_SUBUNIT_BIOGENESIS
RRNA_METABOLIC_PROCESS
TRANSLATION
most shared
most spliced
pathway
NES
0 1 2 3
-log10(padj)
10 20 30
mode
spliced
unspliced
D
Factor 11
SYNCYTIUM_FORMATION
STRIATED_MUSCLE_CONTRACTION
MYOBLAST_FUSION
NEGATIVE_REGULATION_OF_SYNCYTIUM_FORMATION_BY_PLAS...
STRIATED_MUSCLE_CELL_DIFFERENTIATION
RIBOSOMAL_LARGE_SUBUNIT_ASSEMBLY
PROTEIN_RNA_COMPLEX_ORGANIZATION
RIBOSOME_ASSEMBLY
RIBOSOMAL_LARGE_SUBUNIT_BIOGENESIS
RIBOSOMAL_SMALL_SUBUNIT_ASSEMBLY
CENTRAL_NERVOUS_SYSTEM_DEVELOPMENT
INTRINSIC_APOPTOTIC_SIGNALING_PATHWAY_IN_RESPONSE_...
REGULATION_OF_SMALL_GTPASE_MEDIATED_SIGNAL_TRANSDU...
SMALL_GTPASE_MEDIATED_SIGNAL_TRANSDUCTION
SYNAPTIC_SIGNALING
most shared
most spliced
most unspliced
pathway
NES
0 1 2 3
-log10(padj)
0 10 20 30
mode
spliced
unspliced
E
Factor 15
POSITIVE_REGULATION_OF_CYTOKINE_PRODUCTION
REGULATION_OF_CELL_ACTIVATION
GAMMA_DELTA_T_CELL_ACTIVATION
T_CELL_PROLIFERATION
MYELOID_LEUKOCYTE_ACTIVATION
CYTOPLASMIC_TRANSLATION
NEGATIVE_REGULATION_OF_SYNCYTIUM_FORMATION_BY_PLAS...
STRIATED_MUSCLE_CONTRACTION
MUSCLE_CONTRACTION
ANTIMICROBIAL_HUMORAL_RESPONSE
REGULATION_OF_HOMOTYPIC_CELL_CELL_ADHESION
REGULATION_OF_SMALL_GTPASE_MEDIATED_SIGNAL_TRANSDU...
CD4_POSITIVE_ALPHA_BETA_T_CELL_ACTIVATION
NEGATIVE_REGULATION_OF_CELL_ACTIVATION
NEGATIVE_REGULATION_OF_INTRINSIC_APOPTOTIC_SIGNALI...
most shared
most spliced
most unspliced
pathway
NES
0 1 2 3
mode
spliced
unspliced
-log10(padj)
0 1 2 3 4
F
Factor 23
REGULATION_OF_CELL_CELL_ADHESION
NEGATIVE_REGULATION_OF_LYMPHOCYTE_ACTIVATION
REGULATION_OF_B_CELL_ACTIVATION
REGULATION_OF_CELL_ADHESION
REGULATION_OF_T_CELL_ACTIVATION
CYTOPLASMIC_TRANSLATION
STRIATED_MUSCLE_CONTRACTION
EOSINOPHIL_MIGRATION
TRANSLATION
IMMUNE_RESPONSE_TO_TUMOR_CELL
CALCINEURIN_MEDIATED_SIGNALING
DENDRITE_MORPHOGENESIS
POSITIVE_REGULATION_OF_INTERLEUKIN_2_PRODUCTION
REGULATION_OF_ACTIN_FILAMENT_BASED_MOVEMENT
POSITIVE_REGULATION_OF_GLIAL_CELL_PROLIFERATION
most shared
most spliced
most unspliced
pathway
NES
0 1 2 3
mode
spliced
unspliced
-log10(padj)
0 1 2 3
