# Supplementary material for: Joint representation of spliced and unspliced RNA expands information content in single-cell expression data
Source: Bioinform Adv. 2026 Oct 22;6(1):vbag263. doi: 10.1093/bioadv/vbag263 (PMC13718436; doi:10.1093/bioadv/vbag263)
Supplement: Supplementary file 1 [file vbag263_supplementary_data.zip › Table_S1_PBMC.pdf]

Table S1. PBMC epoch-50 held-out test MSE across NMF ranks, modalities, and replicates.

	Added			Spliced Only			Unspliced Only			Concatenated		
k	Rep 1	Rep 2	Rep 3	Rep 1	Rep 2	Rep 3	Rep 1	Rep 2	Rep 3	Rep 1	Rep 2	Rep 3
2	0.0578	0.0576	0.0576	0.0242	0.0242	0.0242	0.0404	0.0404	0.0405	0.0327	0.0327	0.0327
3	0.0558	0.0557	0.0557	0.0237	0.0236	0.0236	0.0391	0.0392	0.0393	0.0318	0.0318	0.0318
4	0.0548	0.0546	0.0546	0.0232	0.0231	0.0231	0.0386	0.0387	0.0388	0.0313	0.0313	0.0312
5	0.054	0.0539	0.0539	0.0228	0.0228	0.0229	0.0384	0.0385	0.0386	0.0308	0.0309	0.0309
6	0.0536	0.0535	0.0536	0.0227	0.0226	0.0226	0.0383	0.0383	0.0384	0.0307	0.0307	0.0307
7	0.0532	0.0531	0.0531	0.0225	0.0225	0.0225	0.0381	0.0382	0.0383	0.0306	0.0305	0.0305
8	0.0529	0.0529	0.0528	0.0224	0.0224	0.0224	0.038	0.0381	0.0382	0.0303	0.0304	0.0304
9	0.0527	0.0526	0.0526	0.0223	0.0223	0.0223	0.0379	0.038	0.0381	0.0302	0.0303	0.0302
10	0.0526	0.0525	0.0525	0.0223	0.0223	0.0223	0.0379	0.0379	0.038	0.0301	0.0302	0.0302
11	0.0525	0.0524	0.0524	0.0223	0.0222	0.0222	0.0378	0.0379	0.038	0.0301	0.0301	0.0301
12	0.0524	0.0523	0.0523	0.0222	0.0222	0.0222	0.0377	0.0378	0.0379	0.0301	0.0301	0.0301
13	0.0523	0.0522	0.0522	0.0222	0.0221	0.0221	0.0377	0.0378	0.0379	0.03	0.0301	0.03
14	0.0522	0.0521	0.0521	0.0221	0.0221	0.0221	0.0377	0.0378	0.0379	0.03	0.03	0.03
15	0.0522	0.0521	0.0521	0.0221	0.0221	0.0221	0.0377	0.0377	0.0378	0.0299	0.03	0.0299
16	0.0521	0.052	0.052	0.0221	0.0221	0.022	0.0376	0.0377	0.0377	0.0299	0.0299	0.0299
17	0.0521	0.052	0.052	0.0221	0.0221	0.022	0.0376	0.0377	0.0377	0.0299	0.0299	0.0299
18	0.052	0.0519	0.0519	0.0221	0.0221	0.022	0.0376	0.0377	0.0377	0.0298	0.0299	0.0299
19	0.052	0.0518	0.0519	0.0221	0.022	0.022	0.0376	0.0377	0.0377	0.0298	0.0299	0.0298
20	0.0519	0.0518	0.0518	0.022	0.022	0.022	0.0375	0.0377	0.0377	0.0298	0.0299	0.0298
21	0.0519	0.0518	0.0518	0.022	0.022	0.022	0.0375	0.0376	0.0377	0.0298	0.0298	0.0298
22	0.0519	0.0518	0.0518	0.022	0.022	0.022	0.0376	0.0376	0.0377	0.0298	0.0298	0.0298
23	0.0518	0.0517	0.0518	0.022	0.022	0.022	0.0376	0.0376	0.0377	0.0298	0.0298	0.0298
24	0.0518	0.0517	0.0517	0.022	0.022	0.022	0.0375	0.0377	0.0377	0.0298	0.0298	0.0298
25	0.0518	0.0517	0.0517	0.022	0.022	0.022	0.0375	0.0377	0.0376	0.0297	0.0298	0.0297
26	0.0518	0.0517	0.0517	0.0264	0.022	0.022	0.0375	0.0377	0.0416	0.0297	0.0298	0.0297
27	0.0518	0.0516	0.0516	0.022	0.022	0.0219	0.0395	0.0382	0.0454	0.0297	0.0298	0.0297
28	0.0517	0.0516	0.0516	0.022	0.022	0.0219	0.0489	0.0379	0.0494	0.0297	0.0298	0.0297
29	0.0517	0.0516	0.0516	0.022	0.022	0.0253	0.0493	0.0382	0.0547	0.0297	0.0297	0.0297
30	0.0517	0.0516	0.0516	0.022	0.022	0.0221	0.045	0.0398	0.0565	0.0297	0.0297	0.0297
31	0.0517	0.0516	0.0516	0.022	0.022	0.0292	0.0377	0.0411	0.06	0.0297	0.0298	0.0297
32	0.0517	0.0516	0.0517	0.022	0.0222	0.024	0.0451	0.0401	0.0484	0.0297	0.0297	0.0297
33	0.0517	0.0515	0.0517	0.0221	0.0221	0.037	0.0386	0.0386	0.0484	0.0297	0.033	0.0297
34	0.0518	0.0515	0.0516	0.0222	0.0221	0.0384	0.0441	0.0395	0.0615	0.0297	0.0357	0.0297
35	0.0517	0.0515	0.0516	0.0222	0.0221	0.0418	0.0437	0.0549	0.0621	0.0297	0.0357	0.0297
36	0.0516	0.0515	0.0515	0.0222	0.0223	0.0375	0.0446	0.0498	0.0685	0.033	0.039	0.0297
37	0.058	0.0515	0.0515	0.0222	0.0223	0.0378	0.0487	0.0698	0.0728	0.0367	0.0373	0.0297
38	0.0549	0.0515	0.0515	0.0224	0.0223	0.0401	0.0551	0.0818	0.0761	0.037	0.0387	0.0297
39	0.0705	0.0515	0.0515	0.0224	0.0223	0.0465	0.0607	0.0787	0.0741	0.0365	0.0382	0.0305
40	0.0711	0.0515	0.0516	0.0224	0.0228	0.0504	0.0622	0.0788	0.0783	0.0297	0.0381	0.0302
41	0.0663	0.0515	0.0515	0.0412	0.0227	0.0452	0.0463	0.0831	0.0856	0.0367	0.036	0.0298
42	0.0518	0.0516	0.0516	0.0588	0.0227	0.0361	0.0523	0.0915	0.0803	0.0348	0.0363	0.0298
43	0.0518	0.0516	0.0516	0.0611	0.0228	0.0319	0.083	0.0879	0.0878	0.0434	0.037	0.0298
44	0.0517	0.0523	0.0516	0.0571	0.0226	0.0312	0.0943	0.1018	0.0942	0.0377	0.036	0.0298
45	0.0519	0.0533	0.0516	0.0539	0.0223	0.0342	0.0896	0.112	0.0921	0.0318	0.0384	0.0297
46	0.0535	0.0537	0.0619	0.0729	0.0229	0.0512	0.0848	0.1154	0.0912	0.0389	0.035	0.0298
47	0.0519	0.0537	0.0657	0.0458	0.0235	0.0382	0.0859	0.1265	0.1134	0.032	0.0298	0.0298
48	0.0519	0.0541	0.0646	0.0498	0.0234	0.0413	0.1177	0.1325	0.1207	0.0301	0.0352	0.0304
49	0.052	0.0559	0.0681	0.0441	0.024	0.0454	0.1117	0.1378	0.1246	0.0302	0.0398	0.0306
50	0.0519	0.0535	0.0724	0.0301	0.0238	0.0436	0.1052	0.1506	0.11	0.0308	0.0422	0.0304
51	0.052	0.0537	0.0761	0.05	0.0245	0.0439	0.1204	0.1349	0.1208	0.0311	0.0431	0.0336
52	0.0524	0.0541	0.0664	0.0495	0.0246	0.0447	0.1269	0.1418	0.1302	0.0314	0.0483	0.0305
53	0.0544	0.0566	0.0731	0.0575	0.026	0.0561	0.1145	0.1558	0.1359	0.031	0.0456	0.0307
54	0.0523	0.0563	0.0822	0.0594	0.0243	0.0895	0.1117	0.16	0.1447	0.0313	0.052	0.0307
55	0.0622	0.0571	0.0825	0.1701	0.0252	0.1324	0.1281	0.1401	0.1664	0.0318	0.0583	0.0307
56	0.0538	0.0567	0.0836	0.133	0.0277	0.1458	0.1875	0.1761	0.1637	0.0334	0.0663	0.0307
57	0.0598	0.0689	0.0909	0.1088	0.0234	0.157	0.1929	0.1711	0.1897	0.033	0.0686	0.0308
58	0.0567	0.075	0.0961	0.0972	0.0251	0.1668	0.195	0.1821	0.1843	0.0335	0.071	0.031
59	0.0955	0.0668	0.0972	0.0944	0.0254	0.1585	0.2145	0.1828	0.1948	0.0345	0.0694	0.0316
60	0.0641	0.0999	0.0995	0.0998	0.0565	0.1452	0.2056	0.1921	0.2902	0.0349	0.0755	0.0315
61	0.0917	0.0697	0.1034	0.1131	0.101	0.1632	0.2369	0.2184	0.3503	0.0352	0.074	0.0319
62	0.0973	0.0717	0.104	0.1011	0.1043	0.1781	0.1923	0.2764	0.3681	0.0404	0.0571	0.0347
63	0.0996	0.0736	0.1124	0.099	0.1152	0.1978	0.1921	0.2798	0.3844	0.0387	0.0882	0.0463
64	0.1196	0.074	0.1142	0.168	0.1257	0.2039	0.2204	0.3073	0.402	0.0436	0.0821	0.0379
65	0.1235	0.0662	0.1132	0.1743	0.1221	0.2114	0.2616	0.3012	0.4472	0.0401	0.0928	0.044
66	0.1189	0.108	0.1075	0.19	0.0627	0.2084	0.2449	0.2504	0.3901	0.0423	0.0907	0.0527
67	0.137	0.1017	0.1077	0.2131	0.1604	0.2254	0.2761	0.2852	0.4327	0.0458	0.0872	0.0541
68	0.1509	0.1029	0.1172	0.2092	0.1751	0.2467	0.3223	0.3353	0.4173	0.0445	0.0898	0.045
69	0.1519	0.1178	0.1158	0.2217	0.1845	0.2857	0.3218	0.3396	0.4241	0.0482	0.0713	0.0437
70	0.1724	0.1176	0.1126	0.2208	0.2005	0.2887	0.3457	0.3525	0.4541	0.0558	0.0707	0.0669
71	0.1898	0.1523	0.0847	0.2257	0.2082	0.2947	0.3673	0.3752	0.4572	0.0554	0.0905	0.0714
72	0.1882	0.1534	0.0955	0.2332	0.2087	0.3091	0.3383	0.309	0.4765	0.056	0.0965	0.1002
73	0.1692	0.1614	0.1008	0.26	0.1659	0.3086	0.3415	0.3428	0.4786	0.0575	0.1161	0.101
74	0.1971	0.1577	0.1203	0.2623	0.1673	0.323	0.4292	0.3768	0.4935	0.0532	0.1076	0.0751
75	0.1896	0.0958	0.1015	0.2543	0.209	0.3429	0.4482	0.3942	0.4619	0.0678	0.109	0.0981
76	0.2097	0.0926	0.1377	0.287	0.2467	0.3716	0.4802	0.3997	0.4404	0.0908	0.1401	0.0936
77	0.1808	0.0912	0.1872	0.3049	0.2551	0.3677	0.5158	0.431	0.5195	0.0922	0.1295	0.0945
78	0.2578	0.105	0.1923	0.3174	0.2552	0.3846	0.511	0.4026	0.5764	0.1018	0.1234	0.1044
79	0.2953	0.1177	0.1964	0.3214	0.2486	0.4172	0.5296	0.4758	0.5369	0.1099	0.1194	0.1098
80	0.2859	0.1378	0.1187	0.3327	0.2713	0.4977	0.5478	0.4579	0.6097	0.1146	0.1271	0.1129

Highlighted cells indicate the selected revealable rank (k*) for each modality and replicate.
