# Supplementary material for: Joint representation of spliced and unspliced RNA expands information content in single-cell expression data
Source: Bioinform Adv. 2026 Oct 22;6(1):vbag263. doi: 10.1093/bioadv/vbag263 (PMC13718436; doi:10.1093/bioadv/vbag263)
Supplement: Supplementary file 1 [file vbag263_supplementary_data.zip › Table_S2_Brain.pdf]

Table S2. Brain epoch-50 held-out test MSE across NMF ranks, modalities, and replicates.

	Added			Spliced Only			Unspliced Only			Concatenated		
k	Rep 1	Rep 2	Rep 3	Rep 1	Rep 2	Rep 3	Rep 1	Rep 2	Rep3	Rep 1	Rep 2	Rep3
2	0.0665	0.0664	0.0665	0.0379	0.0379	0.0379	0.0363	0.0362	0.0362	0.0379	0.038	0.038
3	0.0646	0.0646	0.0646	0.0373	0.0374	0.0373	0.0352	0.0351	0.0351	0.037	0.037	0.037
4	0.0631	0.0631	0.0631	0.0369	0.0369	0.0369	0.0345	0.0344	0.0344	0.0363	0.0363	0.0363
5	0.0618	0.0617	0.0617	0.0366	0.0366	0.0365	0.0341	0.034	0.0341	0.0356	0.0356	0.0356
6	0.0613	0.0612	0.0613	0.0363	0.0363	0.0363	0.0339	0.0338	0.0338	0.0354	0.0354	0.0354
7	0.0608	0.0607	0.0609	0.036	0.036	0.036	0.0337	0.0335	0.0336	0.0352	0.0352	0.0352
8	0.0605	0.0603	0.0605	0.0358	0.0358	0.0358	0.0335	0.0334	0.0334	0.035	0.035	0.035
9	0.0601	0.0601	0.0601	0.0356	0.0357	0.0357	0.0333	0.0332	0.0333	0.0349	0.0348	0.0349
10	0.0598	0.0597	0.0598	0.0355	0.0355	0.0355	0.0332	0.0331	0.0331	0.0347	0.0347	0.0347
11	0.0596	0.0595	0.0595	0.0354	0.0354	0.0354	0.0331	0.033	0.033	0.0346	0.0345	0.0346
12	0.0594	0.0593	0.0593	0.0354	0.0353	0.0354	0.0329	0.0329	0.0329	0.0344	0.0344	0.0344
13	0.0592	0.059	0.0591	0.0353	0.0353	0.0353	0.0328	0.0328	0.0328	0.0343	0.0343	0.0343
14	0.059	0.0589	0.059	0.0352	0.0352	0.0352	0.0328	0.0327	0.0327	0.0342	0.0342	0.0342
15	0.0588	0.0587	0.0588	0.0352	0.0352	0.0351	0.0327	0.0326	0.0326	0.0341	0.0341	0.0342
16	0.0587	0.0586	0.0586	0.0351	0.0351	0.0351	0.0326	0.0325	0.0325	0.0341	0.034	0.0341
17	0.0586	0.0585	0.0586	0.035	0.035	0.035	0.0325	0.0324	0.0324	0.034	0.034	0.034
18	0.0585	0.0584	0.0584	0.035	0.035	0.035	0.0324	0.0324	0.0324	0.0339	0.0339	0.034
19	0.0583	0.0583	0.0583	0.0349	0.0349	0.0349	0.0324	0.0323	0.0323	0.0339	0.0339	0.0339
20	0.0583	0.0582	0.0582	0.0349	0.0349	0.0349	0.0323	0.0323	0.0323	0.0338	0.0338	0.0339
21	0.0582	0.058	0.0581	0.0348	0.0348	0.0349	0.0323	0.0322	0.0322	0.0338	0.0338	0.0338
22	0.058	0.0579	0.058	0.0348	0.0348	0.0356	0.0322	0.0321	0.0322	0.0337	0.0337	0.0337
23	0.058	0.0578	0.058	0.0348	0.0348	0.0348	0.0322	0.0321	0.0321	0.0337	0.0337	0.0337
24	0.0579	0.0578	0.0579	0.0348	0.0348	0.0348	0.0322	0.0321	0.0321	0.0336	0.0336	0.0337
25	0.0578	0.0577	0.0578	0.0349	0.0347	0.0348	0.0321	0.032	0.032	0.0336	0.0336	0.0336
26	0.0577	0.0576	0.0577	0.0349	0.0347	0.0348	0.0321	0.032	0.032	0.0336	0.0336	0.0336
27	0.0577	0.0576	0.0576	0.0483	0.0347	0.0347	0.0321	0.032	0.032	0.0335	0.0335	0.0336
28	0.0576	0.0575	0.0575	0.0357	0.0348	0.0347	0.032	0.032	0.0319	0.0335	0.0335	0.0335
29	0.0575	0.0575	0.0575	0.0503	0.0347	0.0347	0.032	0.0319	0.0319	0.0335	0.0334	0.0335
30	0.0575	0.0574	0.0575	0.0435	0.0347	0.0347	0.032	0.0319	0.0319	0.0334	0.0334	0.0335
31	0.0574	0.0573	0.0574	0.0415	0.0397	0.035	0.032	0.0319	0.0319	0.0334	0.0334	0.0334
32	0.0574	0.0573	0.0573	0.0575	0.0356	0.035	0.0319	0.0319	0.0319	0.0334	0.0334	0.0334
33	0.0573	0.0573	0.0573	0.0659	0.035	0.0347	0.0319	0.0319	0.0319	0.0334	0.0333	0.0334
34	0.0573	0.0572	0.0573	0.0578	0.0352	0.0347	0.0319	0.0318	0.0319	0.0333	0.0333	0.0334
35	0.0572	0.0572	0.0572	0.0652	0.0361	0.0352	0.0319	0.0318	0.0318	0.0333	0.0333	0.0333
36	0.0572	0.0571	0.0572	0.0962	0.0391	0.037	0.0319	0.0318	0.0318	0.0333	0.0333	0.0333
37	0.0572	0.0571	0.0572	0.1084	0.0403	0.0379	0.0319	0.0318	0.0318	0.0333	0.0333	0.0333
38	0.0571	0.0571	0.0571	0.1036	0.0591	0.0411	0.0369	0.0318	0.0318	0.0332	0.0332	0.0333
39	0.0571	0.0571	0.0571	0.1243	0.0837	0.0482	0.0345	0.0318	0.0318	0.0332	0.0332	0.0333
40	0.0571	0.057	0.057	0.1052	0.088	0.0685	0.0352	0.0318	0.0318	0.0332	0.0332	0.0332
41	0.0583	0.057	0.057	0.116	0.0876	0.0739	0.0466	0.0318	0.0319	0.0332	0.0332	0.0332
42	0.0587	0.0572	0.057	0.1009	0.0876	0.0712	0.0394	0.0318	0.0561	0.0345	0.0332	0.0332
43	0.058	0.057	0.057	0.1334	0.0965	0.0974	0.0419	0.0551	0.0622	0.0434	0.0332	0.0332
44	0.0701	0.0577	0.0571	0.1391	0.078	0.0956	0.0326	0.0592	0.0421	0.0405	0.0332	0.0332
45	0.0572	0.0577	0.057	0.1783	0.0846	0.0715	0.0345	0.0789	0.0522	0.0417	0.0333	0.0332
46	0.0585	0.0609	0.0574	0.1641	0.0836	0.1115	0.043	0.0733	0.0482	0.0407	0.0335	0.0388
47	0.0667	0.0647	0.0667	0.1545	0.0971	0.1363	0.0518	0.0733	0.0404	0.0415	0.0344	0.0332
48	0.0696	0.0643	0.0654	0.1643	0.1186	0.139	0.0518	0.077	0.0439	0.0455	0.0357	0.0411
49	0.066	0.0786	0.067	0.1887	0.2129	0.0899	0.0633	0.075	0.05	0.0458	0.0332	0.0393
50	0.0747	0.0588	0.076	0.1663	0.2396	0.1056	0.0798	0.0794	0.0535	0.0332	0.0331	0.0356
51	0.0743	0.0713	0.074	0.1927	0.2528	0.1255	0.0704	0.0905	0.0612	0.0349	0.0332	0.0467
52	0.0747	0.0711	0.0877	0.2261	0.235	0.1479	0.0736	0.1111	0.0589	0.0498	0.0338	0.0539
53	0.0674	0.0762	0.0696	0.2415	0.2687	0.1884	0.0942	0.1595	0.0796	0.0587	0.0385	0.0565
54	0.0737	0.103	0.0849	0.3011	0.2983	0.1871	0.1307	0.1982	0.0627	0.0844	0.047	0.0518
55	0.0787	0.1104	0.0778	0.2892	0.3059	0.1814	0.1131	0.2378	0.1151	0.1071	0.0349	0.0626
56	0.0827	0.0998	0.1003	0.2963	0.3121	0.2033	0.1078	0.2527	0.1194	0.1195	0.0337	0.0613
57	0.0796	0.1066	0.0934	0.3356	0.3203	0.2273	0.1761	0.2756	0.1474	0.1053	0.0345	0.0917
58	0.075	0.1296	0.0941	0.3602	0.351	0.2872	0.1543	0.2324	0.1311	0.1019	0.0374	0.0917
59	0.0737	0.1337	0.0912	0.3601	0.3413	0.3492	0.1637	0.2469	0.1416	0.1014	0.0368	0.0968
60	0.1002	0.1199	0.0976	0.407	0.3776	0.3546	0.2	0.3066	0.1736	0.1362	0.0367	0.0938
61	0.0991	0.138	0.1088	0.3893	0.4061	0.4038	0.2465	0.4119	0.2006	0.1432	0.0359	0.1039
62	0.1148	0.1491	0.1228	0.4422	0.4549	0.4814	0.2222	0.4604	0.2618	0.1398	0.0406	0.1038
63	0.0954	0.1507	0.131	0.4691	0.4724	0.4814	0.2626	0.5093	0.2952	0.1478	0.0412	0.1267
64	0.1225	0.1604	0.1327	0.5866	0.5232	0.4902	0.2645	0.572	0.3027	0.1691	0.0494	0.1309
65	0.1315	0.1541	0.1365	0.6227	0.5607	0.5651	0.3422	0.5363	0.3121	0.167	0.0612	0.1316
66	0.1267	0.162	0.1458	0.6833	0.6158	0.6281	0.3223	0.6858	0.3657	0.1764	0.069	0.1022
67	0.1345	0.1851	0.1386	0.7035	0.6472	0.6208	0.352	0.6866	0.3542	0.1679	0.0646	0.1605
68	0.1403	0.1908	0.1489	0.7165	0.7346	0.6902	0.4498	0.7583	0.3712	0.178	0.0899	0.1685
69	0.1434	0.2133	0.1245	0.8113	0.8543	0.735	0.4824	0.7566	0.385	0.1894	0.1157	0.1768
70	0.1591	0.21	0.1803	0.9153	0.8849	0.7732	0.5266	0.8035	0.4939	0.1938	0.0884	0.1858
71	0.1351	0.2214	0.1808	1.0455	0.8834	0.8488	0.5426	0.8355	0.5174	0.1945	0.1096	0.1959
72	0.197	0.2071	0.1859	1.0713	0.9508	0.8447	0.567	0.9225	0.6162	0.1986	0.1053	0.1906
73	0.2012	0.2062	0.2502	1.1472	0.9871	0.9337	0.5691	0.9747	0.5791	0.1819	0.1068	0.1879
74	0.1994	0.2501	0.2782	1.2015	1.1517	1.0124	0.6385	1.0345	0.662	0.207	0.1437	0.2312
75	0.1928	0.2403	0.3383	1.2573	1.1944	1.081	0.7996	1.0208	0.7081	0.1844	0.149	0.2281
76	0.3012	0.2907	0.3975	1.3221	1.2336	1.1358	0.8148	1.3043	0.8423	0.195	0.1364	0.249
77	0.3254	0.3836	0.3888	1.3812	1.2569	1.1384	0.8355	1.2526	0.8221	0.1924	0.1267	0.2632
78	0.3625	0.3943	0.2948	1.4708	1.3041	1.2114	0.8628	1.2614	0.905	0.2174	0.1511	0.2834
79	0.4081	0.4024	0.2123	1.5062	1.4751	1.2719	0.9255	1.4084	1.0022	0.2659	0.1834	0.2859
80	0.4268	0.4186	0.2458	1.542	1.5874	1.3208	0.9203	1.5088	1.0206	0.2681	0.2171	0.3134

Highlighted cells indicate the selected revealable rank (k*) for each modality and replicate.
