# Supplementary material for: Joint representation of spliced and unspliced RNA expands information content in single-cell expression data
Source: Bioinform Adv. 2026 Oct 22;6(1):vbag263. doi: 10.1093/bioadv/vbag263 (PMC13718436; doi:10.1093/bioadv/vbag263)
Supplement: Supplementary file 1 [file vbag263_supplementary_data.zip › Table_S3_Neuron.pdf]

Table S3. Neuron epoch-50 held-out test MSE across NMF ranks, modalities, and replicates.

	Added			Spliced Only			Unspliced Only			Concatenated		
k	Rep 1	Rep 2	Rep 3	Rep 1	Rep 2	Rep 3	Rep 1	Rep 2	Rep3	Rep 1	Rep 2	Rep3
2	0.0717	0.0717	0.0717	0.0427	0.0428	0.0428	0.0368	0.0368	0.0368	0.0405	0.0406	0.0406
3	0.0698	0.0699	0.0698	0.042	0.0421	0.0421	0.0357	0.0357	0.0358	0.0396	0.0397	0.0397
4	0.0684	0.0684	0.0683	0.0415	0.0416	0.0416	0.0351	0.0351	0.0351	0.0389	0.0389	0.0389
5	0.0671	0.0671	0.0671	0.0411	0.0412	0.0412	0.0347	0.0347	0.0347	0.0383	0.0383	0.0383
6	0.0665	0.0665	0.0664	0.0408	0.0409	0.0409	0.0345	0.0345	0.0345	0.038	0.038	0.0381
7	0.0661	0.0662	0.0661	0.0406	0.0407	0.0407	0.0342	0.0342	0.0343	0.0378	0.0378	0.0378
8	0.0657	0.0657	0.0658	0.0404	0.0405	0.0405	0.0341	0.0341	0.0341	0.0377	0.0377	0.0377
9	0.0654	0.0654	0.0654	0.0402	0.0403	0.0403	0.0339	0.0339	0.034	0.0375	0.0375	0.0375
10	0.065	0.0651	0.0651	0.0401	0.0402	0.0401	0.0338	0.0338	0.0338	0.0373	0.0373	0.0374
11	0.0648	0.0648	0.0648	0.04	0.0401	0.04	0.0337	0.0336	0.0337	0.0372	0.0372	0.0372
12	0.0646	0.0646	0.0646	0.0399	0.04	0.0399	0.0335	0.0335	0.0336	0.0371	0.0371	0.0371
13	0.0644	0.0644	0.0644	0.0398	0.0399	0.0399	0.0335	0.0334	0.0335	0.0369	0.037	0.037
14	0.0642	0.0642	0.0642	0.0397	0.0398	0.0398	0.0333	0.0333	0.0334	0.0369	0.0369	0.0369
15	0.064	0.064	0.064	0.0397	0.0398	0.0397	0.0333	0.0333	0.0333	0.0368	0.0368	0.0368
16	0.0639	0.0639	0.0639	0.0396	0.0397	0.0397	0.0332	0.0332	0.0332	0.0367	0.0367	0.0367
17	0.0638	0.0638	0.0638	0.0396	0.0396	0.0396	0.0331	0.0331	0.0332	0.0366	0.0366	0.0367
18	0.0637	0.0637	0.0637	0.0395	0.0396	0.0396	0.033	0.0331	0.0331	0.0366	0.0366	0.0366
19	0.0635	0.0636	0.0635	0.0394	0.0395	0.0395	0.033	0.033	0.0331	0.0365	0.0365	0.0365
20	0.0635	0.0635	0.0634	0.0394	0.0395	0.0395	0.0329	0.0329	0.033	0.0365	0.0365	0.0365
21	0.0634	0.0634	0.0633	0.0393	0.0394	0.0394	0.0329	0.0329	0.033	0.0364	0.0364	0.0364
22	0.0633	0.0633	0.0633	0.0393	0.0393	0.0394	0.0329	0.0329	0.0329	0.0364	0.0364	0.0364
23	0.0632	0.0631	0.0632	0.0393	0.0393	0.0393	0.0328	0.0328	0.0329	0.0363	0.0363	0.0363
24	0.0631	0.0631	0.0631	0.0392	0.0393	0.0393	0.0328	0.0328	0.0328	0.0363	0.0363	0.0363
25	0.063	0.063	0.063	0.0392	0.0392	0.0393	0.0327	0.0328	0.0328	0.0362	0.0362	0.0362
26	0.0629	0.0629	0.0629	0.0391	0.0392	0.0392	0.0327	0.0327	0.0328	0.0362	0.0362	0.0362
27	0.0628	0.0628	0.0628	0.0391	0.0392	0.0392	0.0327	0.0327	0.0327	0.0362	0.0362	0.0362
28	0.0628	0.0628	0.0628	0.0391	0.0391	0.0392	0.0327	0.0327	0.0327	0.0361	0.0361	0.0361
29	0.0627	0.0627	0.0627	0.039	0.0391	0.0392	0.0326	0.0326	0.0327	0.0361	0.0361	0.0361
30	0.0626	0.0626	0.0626	0.039	0.0391	0.0391	0.0326	0.0326	0.0327	0.036	0.036	0.0361
31	0.0626	0.0626	0.0626	0.039	0.0391	0.0391	0.0326	0.0326	0.0327	0.036	0.036	0.036
32	0.0626	0.0625	0.0625	0.039	0.0391	0.0391	0.0326	0.0326	0.0327	0.036	0.036	0.036
33	0.0624	0.0624	0.0624	0.039	0.039	0.039	0.0326	0.0326	0.0326	0.036	0.0359	0.036
34	0.0624	0.0624	0.0624	0.0389	0.039	0.039	0.0326	0.0326	0.0326	0.0359	0.0359	0.036
35	0.0624	0.0623	0.0624	0.0389	0.039	0.039	0.0325	0.0325	0.0326	0.0359	0.0359	0.0359
36	0.0623	0.0623	0.0623	0.0389	0.039	0.039	0.0325	0.0325	0.0348	0.0359	0.0359	0.0359
37	0.0623	0.0622	0.0622	0.0389	0.039	0.039	0.0325	0.0325	0.0339	0.0358	0.0358	0.0359
38	0.0622	0.0622	0.0622	0.0389	0.039	0.039	0.0325	0.0325	0.0337	0.0358	0.0358	0.0359
39	0.0622	0.0622	0.0621	0.0389	0.039	0.039	0.0325	0.0325	0.033	0.0358	0.0358	0.0358
40	0.0621	0.0621	0.0621	0.0389	0.039	0.039	0.0326	0.0325	0.0335	0.0358	0.0358	0.0358
41	0.0621	0.0621	0.0621	0.0389	0.039	0.039	0.0338	0.0325	0.0328	0.0358	0.0358	0.0358
42	0.0621	0.0621	0.062	0.0389	0.0389	0.039	0.0355	0.0325	0.0331	0.0357	0.0357	0.0358
43	0.0621	0.062	0.062	0.0389	0.0389	0.0389	0.0347	0.0325	0.0327	0.0357	0.0357	0.0358
44	0.062	0.062	0.062	0.0389	0.0389	0.0389	0.0355	0.0326	0.0476	0.0357	0.0357	0.0357
45	0.062	0.062	0.062	0.0389	0.0389	0.0389	0.0396	0.0327	0.034	0.0357	0.0357	0.0357
46	0.0619	0.062	0.0619	0.0389	0.0389	0.0389	0.0419	0.0393	0.04	0.0357	0.0357	0.0357
47	0.0619	0.0619	0.0619	0.0389	0.0389	0.0389	0.0369	0.0565	0.0362	0.0357	0.0357	0.0357
48	0.0619	0.0619	0.0619	0.0389	0.0389	0.0389	0.0393	0.0591	0.0428	0.0357	0.0357	0.0357
49	0.0619	0.0619	0.0619	0.0389	0.0389	0.0389	0.0376	0.0535	0.0477	0.0356	0.0357	0.0357
50	0.0619	0.0619	0.0618	0.0389	0.0389	0.0389	0.0484	0.0747	0.0535	0.0356	0.0356	0.0357
51	0.0618	0.0619	0.0618	0.0389	0.0389	0.0389	0.0644	0.064	0.0404	0.0356	0.0356	0.0356
52	0.0618	0.0619	0.0618	0.0389	0.0389	0.0389	0.0642	0.0521	0.0853	0.0356	0.0356	0.0356
53	0.0618	0.0618	0.0618	0.0389	0.0389	0.0389	0.0517	0.067	0.0893	0.0356	0.0356	0.0356
54	0.0618	0.0618	0.0618	0.0389	0.0389	0.0389	0.0517	0.1087	0.0917	0.0356	0.0356	0.0356
55	0.0618	0.0618	0.0618	0.0389	0.039	0.0389	0.0551	0.0947	0.1171	0.0356	0.0356	0.0356
56	0.0618	0.0618	0.0618	0.0389	0.039	0.0389	0.0606	0.0859	0.1342	0.0356	0.0356	0.0356
57	0.0618	0.0618	0.0617	0.0389	0.0389	0.0389	0.0702	0.1263	0.1116	0.0356	0.0356	0.0356
58	0.0618	0.0617	0.0617	0.0409	0.0389	0.0389	0.0799	0.1017	0.1212	0.0356	0.0356	0.0356
59	0.0617	0.0617	0.0617	0.0419	0.0393	0.0389	0.1121	0.0972	0.1144	0.0356	0.0356	0.0356
60	0.0617	0.0617	0.0617	0.0422	0.039	0.039	0.0943	0.206	0.1226	0.0356	0.0356	0.0356
61	0.0617	0.0617	0.0617	0.0446	0.039	0.039	0.0914	0.2296	0.168	0.0355	0.0356	0.0356
62	0.0617	0.0617	0.0617	0.0417	0.0389	0.0396	0.1466	0.2235	0.1795	0.0355	0.0355	0.0356
63	0.0652	0.0617	0.0617	0.0416	0.0618	0.0399	0.2471	0.2111	0.2042	0.0355	0.0391	0.0356
64	0.0617	0.0617	0.0617	0.0544	0.0577	0.0403	0.2359	0.2464	0.1949	0.0355	0.0371	0.0355
65	0.0617	0.0617	0.0617	0.0601	0.0403	0.039	0.2092	0.2239	0.2217	0.0355	0.039	0.0355
66	0.0617	0.0617	0.0617	0.06	0.039	0.039	0.2665	0.2631	0.2494	0.0355	0.0445	0.0355
67	0.0617	0.0618	0.0616	0.0618	0.0522	0.0391	0.2639	0.3127	0.2099	0.0355	0.0398	0.0355
68	0.0617	0.0619	0.064	0.0608	0.0399	0.0393	0.2702	0.3294	0.2526	0.0355	0.0371	0.0355
69	0.0617	0.0701	0.0625	0.0579	0.0534	0.0454	0.302	0.3123	0.2431	0.0355	0.0421	0.0356
70	0.0617	0.0705	0.0617	0.0675	0.0391	0.039	0.3537	0.3259	0.2759	0.0355	0.0404	0.0355
71	0.0638	0.0691	0.0619	0.0799	0.04	0.0401	0.3951	0.2852	0.2674	0.0355	0.0391	0.0355
72	0.0642	0.066	0.0655	0.0927	0.0446	0.0395	0.3885	0.2707	0.2844	0.0355	0.0385	0.0355
73	0.0718	0.0629	0.0672	0.1025	0.0434	0.0393	0.3651	0.3261	0.3887	0.0356	0.0385	0.0355
74	0.0702	0.0616	0.069	0.1223	0.0518	0.0522	0.398	0.3771	0.4757	0.043	0.0384	0.0358
75	0.0724	0.0616	0.0634	0.1249	0.0505	0.0525	0.4101	0.3591	0.4559	0.0403	0.0373	0.0357
76	0.073	0.0617	0.0704	0.1402	0.0577	0.0534	0.4565	0.372	0.5244	0.047	0.0368	0.0367
77	0.066	0.0635	0.0705	0.1568	0.0684	0.0556	0.5064	0.3957	0.4898	0.0395	0.0355	0.0355
78	0.0806	0.066	0.074	0.1739	0.0923	0.0764	0.5074	0.4629	0.5463	0.0356	0.0375	0.0355
79	0.0777	0.0696	0.0672	0.1816	0.0913	0.0897	0.6033	0.5461	0.5791	0.0356	0.0369	0.0357
80	0.0771	0.0732	0.07	0.1914	0.0944	0.0671	0.6633	0.5012	0.5478	0.0473	0.0366	0.0363

Highlighted cells indicate the selected revealable rank (k*) for each modality and replicate.
