# Supplementary material for: Joint representation of spliced and unspliced RNA expands information content in single-cell expression data
Source: Bioinform Adv. 2026 Oct 22;6(1):vbag263. doi: 10.1093/bioadv/vbag263 (PMC13718436; doi:10.1093/bioadv/vbag263)
Supplement: Supplementary file 1 [file vbag263_supplementary_data.zip › Table_S4_Heart.pdf]

Table S4. Heart epoch-50 held-out test MSE across NMF ranks, modalities, and replicates.

	Added			Spliced Only			Unspliced Only			Concatenated		
k	Rep 1	Rep 2	Rep 3	Rep 1	Rep 2	Rep 3	Rep 1	Rep 2	Rep3	Rep 1	Rep 2	Rep3
2	0.0763	0.0761	0.0763	0.0393	0.0392	0.0392	0.0435	0.0433	0.0434	0.0426	0.0426	0.0426
3	0.0728	0.0726	0.0728	0.0376	0.0377	0.0375	0.0428	0.0426	0.0426	0.0408	0.0408	0.0408
4	0.0713	0.0711	0.0713	0.0365	0.0364	0.0363	0.0422	0.0421	0.0421	0.0401	0.0401	0.0401
5	0.0699	0.0697	0.0697	0.0358	0.0358	0.0357	0.0419	0.0417	0.0417	0.0393	0.0393	0.0392
6	0.0688	0.0686	0.0689	0.0353	0.0352	0.0352	0.0416	0.0414	0.0414	0.0389	0.0389	0.0389
7	0.0682	0.0681	0.0682	0.0349	0.0348	0.0347	0.0413	0.0411	0.0412	0.0385	0.0386	0.0385
8	0.0676	0.0674	0.0676	0.0346	0.0346	0.0345	0.041	0.0409	0.041	0.0383	0.0384	0.0383
9	0.0672	0.067	0.0673	0.0343	0.0343	0.0342	0.0409	0.0407	0.0408	0.038	0.0381	0.0381
10	0.0668	0.0666	0.0669	0.0341	0.0341	0.034	0.0407	0.0406	0.0407	0.0378	0.0379	0.0378
11	0.0663	0.0662	0.0664	0.034	0.0339	0.0338	0.0406	0.0404	0.0405	0.0377	0.0376	0.0376
12	0.066	0.0659	0.0661	0.0337	0.0337	0.0336	0.0405	0.0403	0.0404	0.0375	0.0375	0.0375
13	0.0657	0.0656	0.0657	0.0336	0.0335	0.0335	0.0404	0.0402	0.0402	0.0373	0.0374	0.0373
14	0.0654	0.0653	0.0656	0.0334	0.0334	0.0334	0.0403	0.0402	0.0402	0.0372	0.0372	0.0372
15	0.0651	0.0651	0.0653	0.0333	0.0332	0.0332	0.0402	0.0401	0.0401	0.037	0.0371	0.0371
16	0.0649	0.0648	0.0651	0.0332	0.0332	0.0331	0.0402	0.04	0.04	0.0369	0.037	0.0369
17	0.0647	0.0647	0.0649	0.033	0.033	0.0329	0.0401	0.04	0.04	0.0368	0.0368	0.0368
18	0.0646	0.0644	0.0647	0.033	0.0329	0.0329	0.0401	0.0399	0.0399	0.0368	0.0368	0.0367
19	0.0644	0.0643	0.0645	0.0329	0.0328	0.0328	0.0401	0.0398	0.0399	0.0367	0.0367	0.0367
20	0.0643	0.0642	0.0644	0.0328	0.0328	0.0327	0.04	0.0398	0.0398	0.0366	0.0366	0.0366
21	0.0642	0.0641	0.0643	0.0327	0.0327	0.0327	0.04	0.0398	0.0398	0.0365	0.0366	0.0365
22	0.0641	0.064	0.0642	0.0327	0.0326	0.0326	0.0399	0.0398	0.0398	0.0365	0.0365	0.0365
23	0.064	0.0639	0.0641	0.0327	0.0326	0.0325	0.0399	0.0398	0.0398	0.0364	0.0364	0.0364
24	0.0639	0.0638	0.064	0.0326	0.0325	0.0325	0.0399	0.0397	0.0397	0.0364	0.0364	0.0364
25	0.0639	0.0637	0.0639	0.0325	0.0325	0.0324	0.0399	0.0397	0.0397	0.0363	0.0364	0.0363
26	0.0638	0.0636	0.0638	0.0325	0.0325	0.0324	0.0399	0.0397	0.0397	0.0363	0.0363	0.0363
27	0.0637	0.0636	0.0638	0.0324	0.0324	0.0324	0.0399	0.0397	0.0397	0.0362	0.0363	0.0363
28	0.0637	0.0635	0.0637	0.0324	0.0324	0.0324	0.0399	0.0397	0.0397	0.0362	0.0362	0.0362
29	0.0636	0.0634	0.0637	0.0324	0.0324	0.0323	0.0398	0.0397	0.0397	0.0362	0.0362	0.0362
30	0.0635	0.0634	0.0636	0.0324	0.0325	0.0323	0.0398	0.0397	0.0397	0.0361	0.0362	0.0361
31	0.0634	0.0633	0.0635	0.0323	0.0324	0.0323	0.0398	0.0397	0.0397	0.0361	0.0361	0.0361
32	0.0634	0.0633	0.0635	0.034	0.0323	0.0324	0.0398	0.0397	0.0397	0.0361	0.0361	0.0361
33	0.0634	0.0632	0.0634	0.0323	0.0323	0.0325	0.0398	0.0397	0.0397	0.0361	0.0361	0.0361
34	0.0633	0.0632	0.0634	0.0325	0.0324	0.0371	0.0398	0.0396	0.0397	0.036	0.0361	0.036
35	0.0633	0.0632	0.0634	0.0324	0.0325	0.0329	0.0398	0.0396	0.0397	0.036	0.0361	0.036
36	0.0632	0.0632	0.0633	0.0327	0.0328	0.033	0.0398	0.0397	0.0397	0.036	0.036	0.036
37	0.0632	0.0631	0.0633	0.0329	0.0324	0.0429	0.0398	0.0396	0.0396	0.036	0.036	0.036
38	0.0631	0.0631	0.0632	0.0328	0.0332	0.0469	0.0398	0.0396	0.0397	0.036	0.036	0.0359
39	0.0632	0.063	0.0632	0.0368	0.0352	0.0536	0.0398	0.0397	0.0397	0.0359	0.036	0.0359
40	0.0631	0.063	0.0632	0.0367	0.0397	0.0597	0.0398	0.0397	0.0527	0.0359	0.036	0.0359
41	0.0631	0.063	0.0632	0.0381	0.0452	0.0706	0.0398	0.0397	0.0528	0.0359	0.036	0.0359
42	0.063	0.0629	0.0632	0.0422	0.0505	0.0829	0.0398	0.0397	0.0481	0.0359	0.0364	0.0359
43	0.063	0.0629	0.0631	0.039	0.048	0.0828	0.0398	0.0397	0.0397	0.0359	0.0359	0.0378
44	0.063	0.0629	0.0631	0.0634	0.0492	0.0831	0.0398	0.0397	0.0397	0.0359	0.0359	0.0359
45	0.063	0.0629	0.0634	0.0715	0.0613	0.0831	0.0398	0.0397	0.0397	0.0359	0.0376	0.0359
46	0.063	0.063	0.0634	0.0624	0.055	0.0881	0.0442	0.0397	0.0397	0.0369	0.0359	0.0365
47	0.063	0.0642	0.0632	0.0674	0.054	0.0723	0.0552	0.0397	0.0397	0.0373	0.0359	0.0362
48	0.063	0.0652	0.0634	0.0901	0.0674	0.0601	0.0575	0.0397	0.0397	0.038	0.0359	0.0358
49	0.0729	0.0645	0.0663	0.0996	0.1388	0.0819	0.0603	0.0397	0.0397	0.036	0.0359	0.0358
50	0.0736	0.0646	0.063	0.0994	0.123	0.0874	0.0586	0.0424	0.0397	0.0358	0.0358	0.0358
51	0.0629	0.0649	0.0771	0.1195	0.0826	0.0866	0.0605	0.0435	0.0397	0.0365	0.0358	0.0358
52	0.0657	0.0651	0.0632	0.1024	0.0855	0.1074	0.0604	0.047	0.0419	0.0358	0.0359	0.0358
53	0.0665	0.0984	0.0739	0.102	0.0868	0.102	0.0595	0.0501	0.0398	0.037	0.0358	0.0358
54	0.0686	0.0906	0.0743	0.1001	0.083	0.1259	0.0669	0.0543	0.0398	0.0359	0.0358	0.0358
55	0.0759	0.0951	0.0938	0.1049	0.0827	0.1275	0.0638	0.0526	0.0399	0.0373	0.0376	0.0388
56	0.063	0.0858	0.0921	0.1044	0.1747	0.1143	0.0776	0.0552	0.0685	0.0358	0.0376	0.0407
57	0.0698	0.0838	0.0836	0.0984	0.1965	0.1227	0.084	0.058	0.1284	0.0358	0.0469	0.0395
58	0.0851	0.0827	0.0892	0.1349	0.2036	0.1444	0.0952	0.0583	0.0777	0.0361	0.0459	0.0402
59	0.0956	0.0832	0.0881	0.1516	0.1817	0.1639	0.0936	0.0584	0.1157	0.036	0.0463	0.0431
60	0.0779	0.0795	0.0944	0.1781	0.1875	0.145	0.1066	0.064	0.1348	0.0359	0.0469	0.0423
61	0.0739	0.0875	0.09	0.1684	0.1756	0.1561	0.1124	0.0748	0.0848	0.0378	0.0521	0.0411
62	0.0817	0.0921	0.0882	0.1758	0.1886	0.1514	0.1101	0.0817	0.0833	0.0377	0.051	0.0438
63	0.0852	0.0872	0.0805	0.2012	0.2104	0.1974	0.1131	0.0795	0.0893	0.0364	0.0666	0.045
64	0.0994	0.0885	0.0905	0.1975	0.2136	0.2136	0.1504	0.0841	0.0718	0.0402	0.0514	0.0424
65	0.0981	0.0914	0.0784	0.1877	0.1977	0.2033	0.1666	0.0886	0.1352	0.0416	0.0511	0.0603
66	0.1002	0.1028	0.0705	0.1733	0.1993	0.2317	0.263	0.0967	0.144	0.0395	0.0508	0.0514
67	0.0918	0.1022	0.0732	0.1772	0.2065	0.2777	0.2885	0.1008	0.1653	0.0448	0.0512	0.0488
68	0.0822	0.079	0.0784	0.1966	0.2053	0.31	0.3108	0.1088	0.1333	0.0475	0.0588	0.0546
69	0.082	0.1074	0.0826	0.1924	0.2281	0.3151	0.3437	0.1071	0.1923	0.0377	0.0615	0.0493
70	0.0915	0.1126	0.0975	0.224	0.2791	0.2742	0.3975	0.105	0.1872	0.0455	0.0676	0.0553
71	0.0932	0.1085	0.102	0.2228	0.2892	0.3196	0.4555	0.0991	0.1945	0.0391	0.0601	0.0611
72	0.0933	0.1059	0.103	0.3124	0.323	0.284	0.4901	0.1626	0.2086	0.0703	0.0912	0.0622
73	0.1849	0.1077	0.1084	0.3053	0.3722	0.3692	0.4962	0.1766	0.2304	0.0729	0.0743	0.0649
74	0.1537	0.1068	0.1151	0.2955	0.379	0.4314	0.5388	0.202	0.2619	0.0727	0.0758	0.0595
75	0.1585	0.107	0.1168	0.3028	0.378	0.4756	0.573	0.2639	0.3375	0.0688	0.0755	0.0615
76	0.1828	0.1038	0.126	0.3427	0.372	0.4764	0.6183	0.2867	0.3446	0.0653	0.077	0.0654
77	0.2089	0.1148	0.1273	0.4541	0.3899	0.4544	0.6576	0.3278	0.3618	0.0711	0.0788	0.0563
78	0.2158	0.1242	0.102	0.471	0.4325	0.4727	0.7284	0.3665	0.4174	0.0687	0.0689	0.0711
79	0.2015	0.1072	0.1291	0.4708	0.5156	0.5203	0.6915	0.4287	0.4553	0.0711	0.075	0.0726
80	0.2013	0.1182	0.1209	0.4876	0.4859	0.5448	0.7495	0.4696	0.5645	0.0697	0.0676	0.076

Highlighted cells indicate the selected revealable rank (k*) for each modality and replicate.
